# Machine learning analysis of pregnancy data enables early identification of a subpopulation of newborns with ASD

**DOI:** 10.1038/s41598-021-86320-0

**Published:** 2021-03-25

**Authors:** Hugues Caly, Hamed Rabiei, Perrine Coste-Mazeau, Sebastien Hantz, Sophie Alain, Jean-Luc Eyraud, Thierry Chianea, Catherine Caly, David Makowski, Nouchine Hadjikhani, Eric Lemonnier, Yehezkel Ben-Ari

**Affiliations:** 1 Gynecology-Obstetrics Department, Mère-Enfant Hospital, University Hospital Center, Limoges, France; 2 BABiomedical, Luminy Scientific Campus, Marseille, France; 3 Neurochlore, Luminy Scientific Campus, Marseille, France; 4 Bacteriology-Virology-Hygiene Department, University Hospital Center, Limoges, France; 5 French National Reference Center for Herpes Viruses, University Hospital Center, Limoges, France; 6 Department of Biochemistry and Molecular Genetics, Dupuytren University Hospital, Limoges, France; 7 INRAE, UMR MIA 518, INRA AgroParisTech Université Paris-Saclay, Paris, France; 8 Martinos Center for Biomedical Imaging, Harvard Medical School, Boston, USA; 9 Gillberg Neuropsychiatry Center, Sahlgrenska Academy, Gothenburg University, Gothenburg, Sweden; 10 Autism Expert Center and Autism Resource Center of Limousin, University Hospital Center, Limoges, France

**Keywords:** Machine learning, Data mining, Statistical methods, Autism spectrum disorders

## Abstract

To identify newborns at risk of developing ASD and to detect ASD biomarkers early after birth, we compared retrospectively ultrasound and biological measurements of babies diagnosed later with ASD or neurotypical (NT) that are collected routinely during pregnancy and birth. We used a supervised machine learning algorithm with a cross-validation technique to classify NT and ASD babies and performed various statistical tests. With a minimization of the false positive rate, 96% of NT and 41% of ASD babies were identified with a positive predictive value of 77%. We identified the following biomarkers related to ASD: sex, maternal familial history of auto-immune diseases, maternal immunization to CMV, IgG CMV level, timing of fetal rotation on head, femur length in the 3rd trimester, white blood cell count in the 3rd trimester, fetal heart rate during labor, newborn feeding and temperature difference between birth and one day after. Furthermore, statistical models revealed that a subpopulation of 38% of babies at risk of ASD had significantly larger fetal head circumference than age-matched NT ones, suggesting an in utero origin of the reported bigger brains of toddlers with ASD. Our results suggest that pregnancy follow-up measurements might provide an early prognosis of ASD enabling pre-symptomatic behavioral interventions to attenuate efficiently ASD developmental sequels.

## Introduction

Autism Spectrum Disorder (ASD) is characterized by persistent communication and social interactions deficits, and restricted, repetitive behaviors (DSM-5—APA 2013)^1,2^. Since the first studies in the 1960s, its prevalence has steadily increased from 0.041 to 1.68%^3^. This increase is due to modifications of diagnostic criteria, wider access to diagnosis, and a genuine increase due to a combination of genetic and environmental components^4,5^. In spite of the incidence of autism, there is yet no FDA or EMA approved drug agent to treat its core symptoms.

Clinical and histological observations suggest that ASD is generated in the womb. Thus, increased ASD incidence has been related to maternal viral or microbial infection, febrile episodes, activation of the immune system^6–9^, drugs taken during pregnancy, notably sodium valproate^10^, deficiency of Vitamin D^11^, or exposure to environmental hazards^12,13^. Post-mortem analysis of brains from children with ASD reveals an abnormal excess of neurons in the prefrontal cortex indicative of an in utero origin^14^. Furthermore, brain overgrowth and megalencephalic brains have been reported in a subpopulation of children and adolescents with ASD^15,16^, but whether this is initiated already in utero remains controversial^17–21^. The incidence of ASD is also increased by C-Section delivery, obstetric complications and preterm birth^22,23^.

Experimental data also suggest an in utero pathogenesis of ASD^24–29^. Thus, Maternal Immune Activation (MIA) or maternal valproate administration during gestation are associated with ASD^25,30^. Also, early post-natal alterations are observed in the ASD in utero VPA model and in Fragile X mice^31^. Brain overgrowth during parturition and birth has been observed in the rodent ASD in utero VPA model^30^. Collectively, these observations illustrate the extreme heterogeneity of pathological events associated with ASD and the need to identify the sequence of events generated by the initial in utero insult.

Here, we analyzed biological and ultrasound measurements routinely collected in maternities from the first pregnancy trimester to 1 day after birth to determine if they could enable a prognosis of ASD at birth. To this aim, we compared retrospectively maternity parameters in babies diagnosed 4–5 years later with ASD, and in an age-matched population of neurotypical (NT) babies. Due to the large number of features and complex multivariate and poorly understood links between them, we used several statistical tools to reveal patterns that distinguish NT babies from those with ASD.

A supervised machine learning (ML) algorithm was trained to classify babies in two groups, ASD and NT. A cross-validation (CV) technique was used to ensure the generalizability of the classifier’s results on an unseen independent cohort. The features with the highest impact on the classifier’s decisions were identified and analyzed more precisely. In parallel, significant changes in the distribution of all collected features between NT and ASD babies were identified through conventional statistical hypothesis tests. Finally, the longitudinal developmental trajectories of head circumference (HC) growth in fetuses were analyzed by statistical models to investigate the possibility that megalencephalic ASD brains in children and adolescents are generated in utero.

The use of follow-up features routinely collected in maternities without expensive additional tests will allow for an early prognosis of ASD and therefore facilitate the start of early behavioral treatments, known to be more efficient when initiated before the end of the developmental plasticity period^32,33^.

## Results

A classifier was trained on our dataset with a strategy aimed at minimizing false ASD detections while keeping true ASD detection rate as high as possible. The performance of the classifier was evaluated by the estimation of classification scores through a CV process. To reduce the risk of overfitting and assess the ability of the classifier to act without human intervention, two feature selection strategies (FSS) were employed.

i) We first used a semi-automatic FSS with a preselection of features relying on domain knowledge and observations that suggest an implication of a given feature in ASD pathogenesis. With this approach, the true negative rate, i.e. the proportion of NT babies correctly classified as NT, was of 0.96 (95% CI = [0.95, 0.97]), thus only 4% of NT babies were wrongly classified as ASD (Table 1). The true positive rate, i.e. the proportion of babies with ASD correctly classified in the ASD group, was of 0.41 (95% CI = [0.37, 0.45]). However, the positive predictive value was as high as 0.77 (95%CI = [0.72, 0.81]), implying that 77% of babies classified as ASD were indeed diagnosed later as babies with ASD.

**Table 1:** Evaluation of the classifier performance.

FS strategy	TNR	TPR	PPV	F_0.5_
Semi-automatic	0.96 ± .01	0.41 ± .04	0.77 ± .05	0.62 ± .04
Fully automatic	0.96 ± .01	0.41 ± .04	0.77 ± .05	0.63 ± .04

Estimated classification scores with 95% confidence intervals computed through averaging on a cross-validation process based on two feature selection strategies (FSS). TNR true negative rate, TPR true positive rate, PPV positive predictive value.

ii) We then used an automatic FSS that does not rely on medical presumptions on links between features and ASD pathogenesis. By using this strategy, the classifier achieved the same performance as with the semi-automatic FSS (Table 1).

Therefore, NT babies were almost completely correctly identified at birth and a prognostic of ASD could be made in a subgroup of babies with a high precision. Moreover, the classifier can cope efficiently with a large feature space without any medical presumptions.

### Identification of important features

To identify features that play an important role in the classification process, we considered two approaches.

### Feature frequency in the cross-validation folds

**Figure 1:**
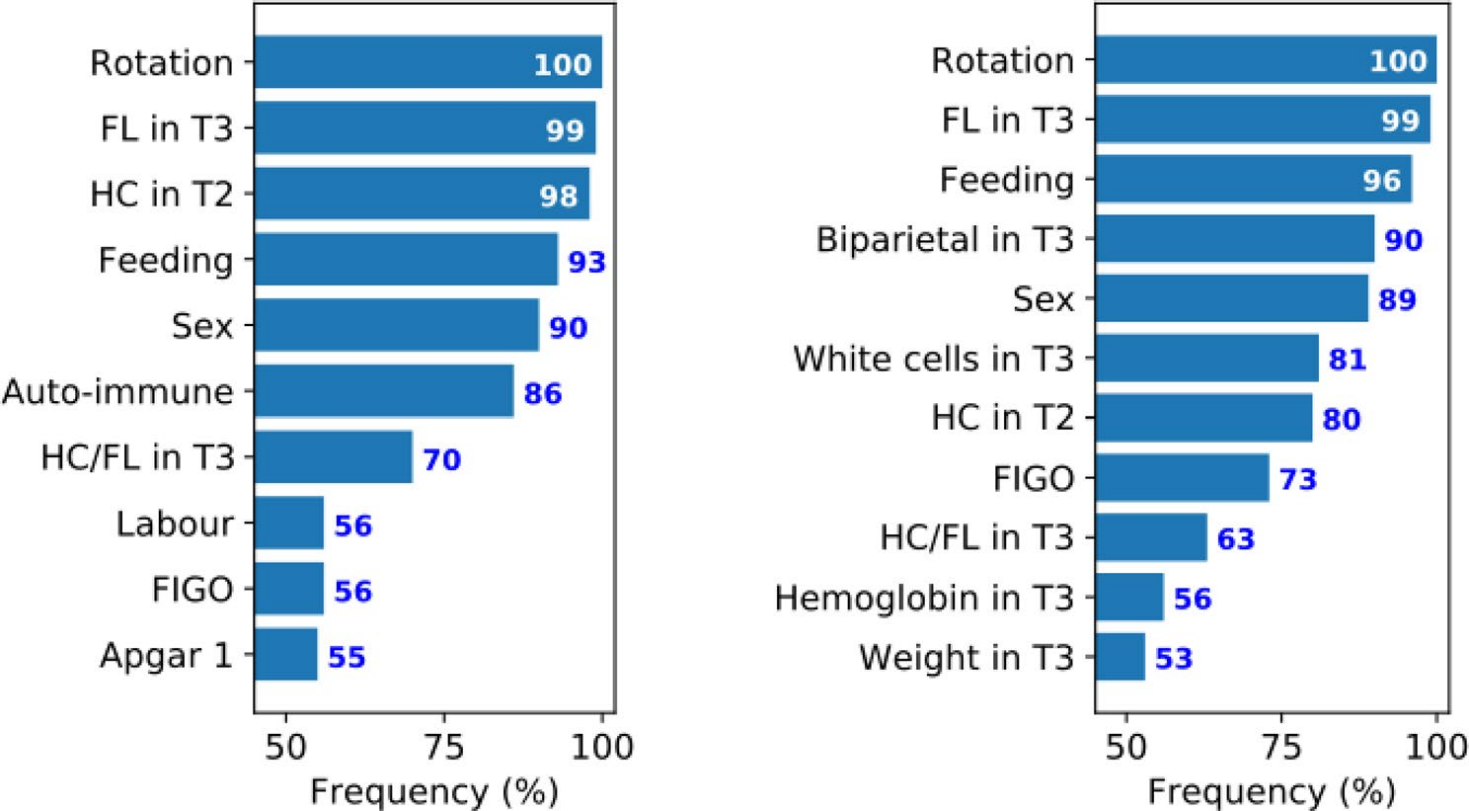
Identification of biomarkers through feature frequency in the cross-validation process. Features that have been selected by the semi-automatic (left plot) and automatic (right plot) feature selection strategies in more than 50 out of 100 folds of the CV process. Features with higher frequencies are more important to the classifier which means they separate better NT babies from ASD ones. T2, second trimester; T3, third trimester; Rotation, timing of fetal rotation on head (days); FL, femur length percentile; HC, head circumference percentile; Feeding, newborn feeding; Auto-immune, familial maternal history of auto-immune diseases; HC/FL, ratio of head circumference to femur length; Labor, duration of the first part of the labour; FIGO, fetal heart rate during labor (FIGO classification); Apgar 1, Apgar score in 1 min; Biparietal, biparietal diameter percentile; Weight, fetal weight percentile estimation.

In the cross-validation (CV) process, the classifier is trained from scratch in each fold and selects features that distinguish better NT from ASD babies in the training set. Features that have been selected by the classifier in at least half of the CV folds are given in Fig. 1. In both automatic and semi-automatic FSSs the timing of fetal rotation on head, femur length percentile in the 3rd trimester (T3), head circumference percentile in the 2nd trimester (T2), newborn feeding, sex, ratio of head circumference to femur length in T3, and fetal heart rate during labor (FIGO classification) were considered as important factors.

Other features were more frequent in one FSS than the other. Thus, in the semi-automatic FSS, familial maternal history of auto-immune diseases, duration of the first part of the labor and Apgar score in 1 min appeared frequently. In the automatic FSS, biparietal diameter in T3, white blood cell count in T3, hemoglobin in T3 and fetal weight estimation in T3 were important features. In other words, the classifier with automatic FSS could detect patterns in some features that are not normally considered relevant to ASD. Some features were selected unfrequently and many others considered as irrelevant by the classifier and thus never been selected. A complete list of features selected by the classifier at least once in the CV process is shown in Supplementary Table S1.

### Feature impacts

**Figure 2:**
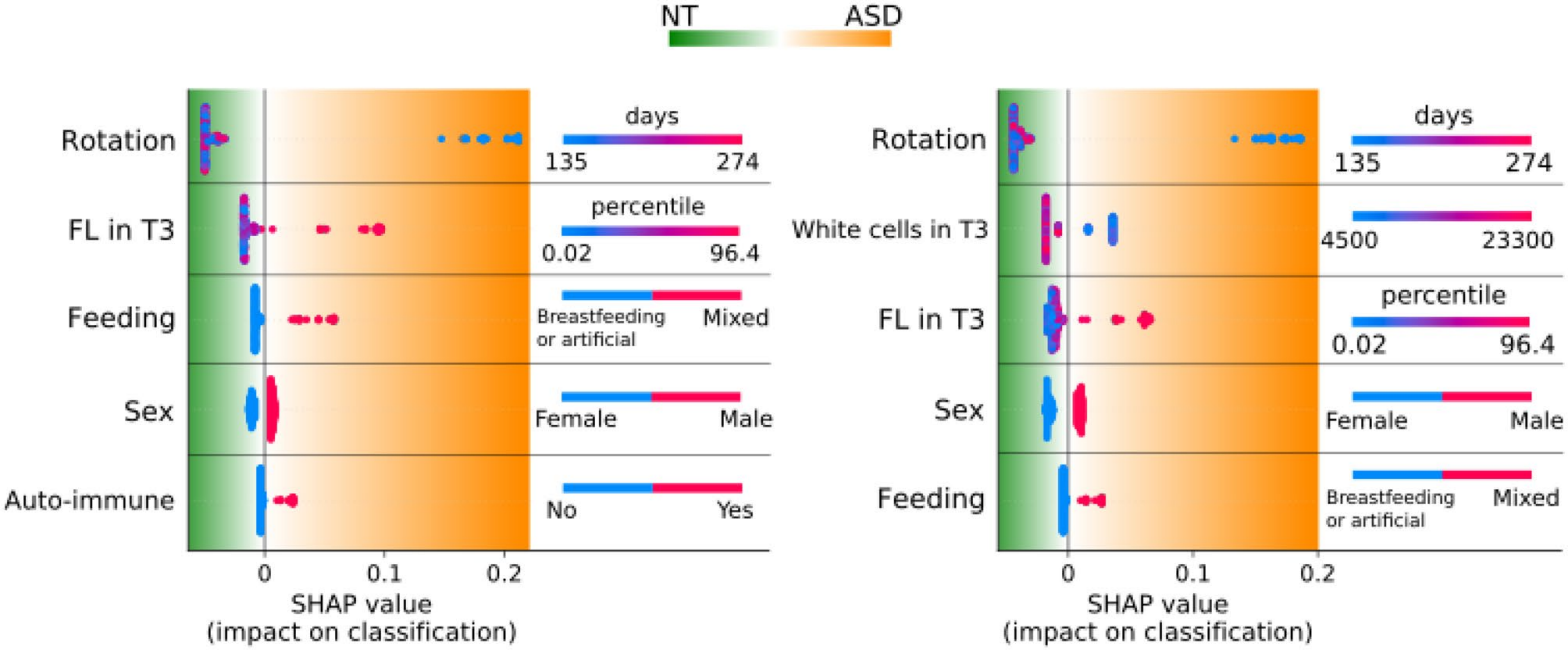
Features with the highest impact on the classifier based on SHAP analysis. Left: classification with the semi-automatic FSS. Right: classification with the automatic FSS. The impact (SHAP values) of the features on the classification as NT or ASD is shown. The features are ordered from top to bottom according to their decreasing impact. Each point at each feature line represents a baby colored by the corresponding feature value. In each plot, the feature values that lead the classifier to an NT or ASD prognosis are on the green and orange regions, respectively. T3, third trimester; Rotation, timing of fetal rotation on head; Feeding, newborn feeding; FL, femur length percentile; Auto-immune, familial maternal history of auto-immune diseases.

Our second approach to identify important features relies on the Shapley additive explanations (SHAP) framework. This computes the impact of each feature on the classifier’s output and the range of values of each feature to increase the probability of babies to be classified as NT or ASD. SHAP values of 5 features with the highest relative impact are shown in Fig. 2 for all babies for both FSSs. In each feature line, a point colored by the corresponding feature value represents one baby and the color map indicates how each feature’s impact varies according to its values. Feature values situated in the positive or negative SHAP side (orange or green regions) leads to ASD or NT classification, respectively. The features with the highest impact and the range of values that push the classifier to the ASD decision are shown in Table 2.

**Table 2:** Features with the highest impact on the classifier based on SHAP analysis.

Feature	Relative impact (%)	Alarming range
**A. Semi-automatic feature selection strategy**		
Rotation	52	Earlier than 148 days
FL in T3	19	Higher than 72%
Newborn feeding	9	Mixed (breastfeeding and artificial)
Sex	7	Male
Auto-immune	4	Yes
**B. Fully automatic feature selection strategy**		
Rotation	44	Earlier than 148 days
White cells in T3	16	Less than 9100
FL in T3	13	Higher than 72%
Sex	9	Male
Newborn feeding	5	Mixed (breastfeeding and artificial)

For each feature, the relative impact and the alarming range, i.e. the range of values that push the classifier to ASD decision, are presented. T3, third trimester. Rotation, timing of fetal rotation on head; FL, femur length percentile; Auto-immune, familial maternal history of auto-immune diseases.

Thus, in both feature selection strategies, fetuses who rotated on their head before 148 days of gestational age were more likely to be classified in the ASD group. In fact, statistical analysis shows 35.09% of ASD babies rotated earlier than 148 days, which is significantly different from 3.72% of NT ones who rotated in that period (Chi-sq = 40.74, *p* < 0.001, df = 1, N = 245). Also, high values of femur length percentile in T3 (higher than 72%) led to an ASD prognostic by the classifier. The proportion of ASD babies with femur length percentile larger than 72% in T3 is 24.56% whereas 7.94% of NT babies have a large femur length in this range (Chi-sq = 10.10, *p* = 0.001, df = 1, N = 246). Feeding babies with a mixture of breast and artificial milk led to an ASD classification. Indeed, 17.86% of ASD babies were fed in a mixed way compared to 5.91% in the NT group (Chi-sq = 6.31, *p* = 0.01, df = 1, N = 242). Not surprisingly, boys were more likely to be classified as ASD than girls. In the ASD group, 80.95% of babies are male whereas the proportion of males in the NT group is almost balanced with 48.68% (Chi-sq = 18.76, *p* < 0.001, df = 1, N = 252).

Familial maternal history of auto-immune diseases was considered an important feature by the semi-automatic FSS. The proportion of ASD babies with familial maternal history of auto-immune diseases is about 19.05%, whereas the proportion of NT babies with this feature is 6.35% (Chi-sq = 4.73, *p* = 0.006, df = 1, N = 252). For the automatic FSS, a white blood cell count lower than 9100 in T3 led the classifier to an ASD decision. Among ASD babies, 47.54% of them have a white blood cell count less than 9100 whereas the proportion of NT babies having this cell count is about 27.65% (Chi-sq = 7.17, *p* = 0.007, df = 1, N = 231). A complete list of features with nonzero relative impact is given in the Supplementary Table S1.

Correlational analysis did not show any moderate to high correlation between features identified by the SHAP framework and other features (Supplementary Table S2). In other words, a feature correlation does not have a significant impact on the classifier’s outputs. It implies that the potential relationship between any extracted feature and ASD development cannot be explained by another feature.

### Statistical difference in feature distributions between ASD and NT

**Figure 3:**
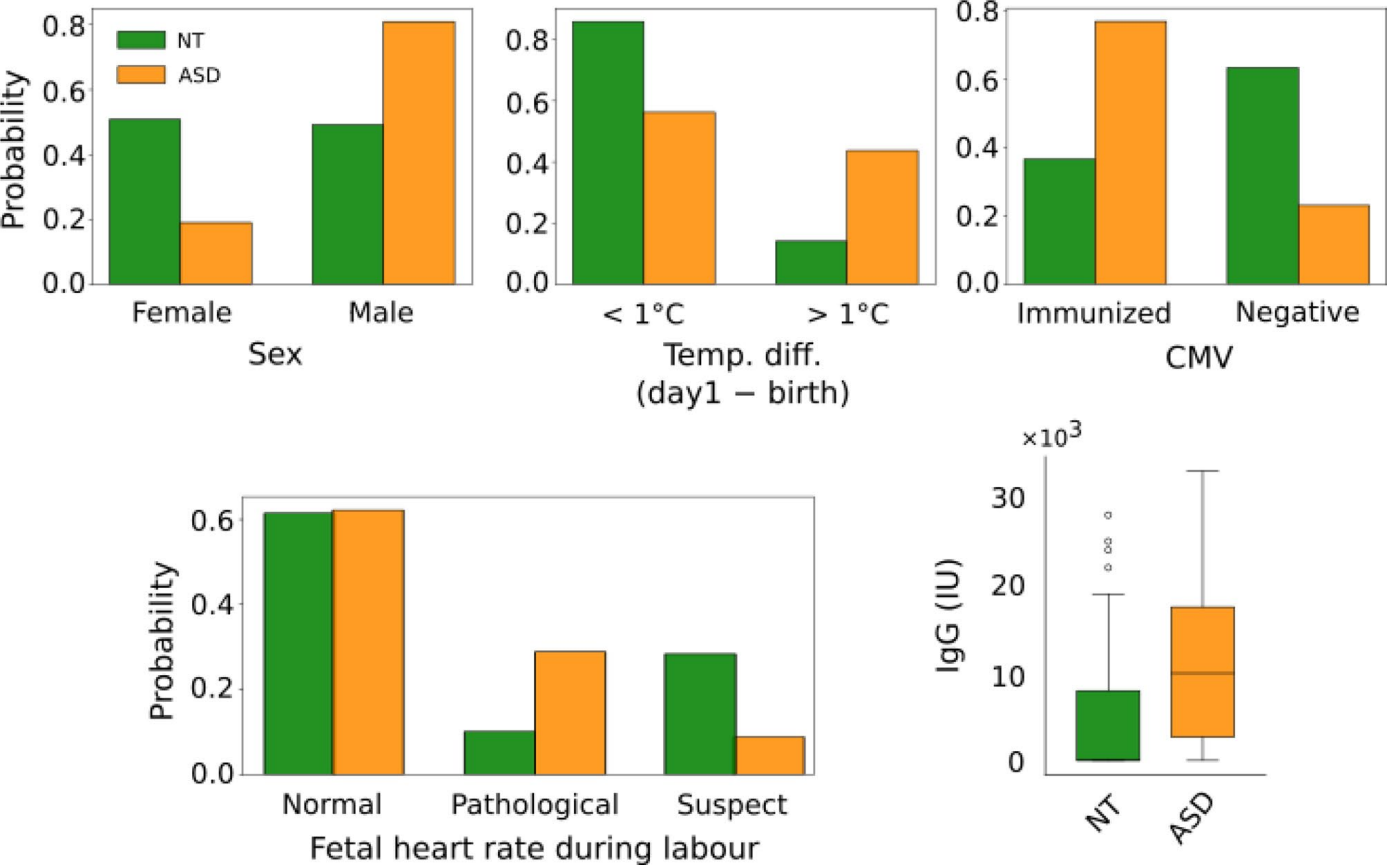
Normalized distribution of statistically significant features. Distributions of Sex, Absolute child’s temperature difference between birth and 1 day later, CMV immunoreactivity, Fetal heart rate during labour (FIGO classification) and IgG levels are shown in the NT (green) and ASD (orange) groups. In the boxplot the center line is the median, the box limits are upper and lower quartiles, and the whiskers are the 1.5 × interquartile range. See Table 3 for quantitative comparisons.

Using conventional statistical tests on all recorded features, the results for features with significantly different distributions in NT and ASD groups are presented in Table 3 and Fig. 3, and in the Supplementary Table S3 for all other features. In the case of categorical features, the number (n) and frequency (%) of babies in each group and the results of the Chi-squared test (Chi-sq) are given. For the numerical features, the number of samples, median, mean, standard error of the mean (SEM) and 95% confidence interval of the mean of feature values in each group together with results of Mann–Whitney U test (MWU) are presented.

**Table 3:** Results of the statistical tests for features which are significantly different between NT and ASD groups.

Feature	Statistics		
	NT group	ASD group	Test results
**Sex**			
Male % (n)	48.68 (92)	80.95 (51)	Chi-sq = 18.76, *p* < 0.001, df = 1, N = 252
Female % (n)	51.32 (97)	19.05 (12)	
**Absolute child’s temperature difference day 1 – birth**			
> 1 °C % (n)	14.00 (21)	41.67 (20)	Chi-sq = 15.31, * p* < 0.001, df = 1, N = 198
< 1 °C % (n)	86.00 (129)	58.33 (28)	
**CMV**			
Immunized % (n)	36.62 (26)	76.92 (20)	Chi-sq = 10.83, * p* < 0.001, df = 1, N = 97
Negative % (n)	63.38 (45)	23.08 (6)	
**Fetal heart rate during labour (FIGO classification)**			
Pathological % (n)	10.20 (15)	28.89 (13)	Chi-sq = 13.82, * p* = 0.001, df = 2, N = 192
Suspect % (n)	28.57 (42)	8.89 (4)	
Normal % (n)	61.22 (90)	62.22 (28)	
**IgG (IU)**			
n	64	22	MWU = 382.5 * p* < 0.001
Median	0	9950	
Mean	4675	10,913.64	
SEM	963.19	2034	
95% CI	[2750.23, 6599.77]	[6683.70, 15,143.57]	

n, number of samples; SEM, standard error of mean; CI, confidence interval; Chi-sq, Chi-squared test; MWU, statistics of Mann–Whitney U test.

Among NT babies, 48.68% are male versus 80.95% of ASD (Chi-sq = 18.76, *p* < 0.001, df = 1, N = 252). There was a temperature difference of more than 1 °C (in either direction) between birth and day 1 in 14% and 41.67% of NT and ASD newborns respectively (Chi-sq = 15.31, *p* < 0.001, df = 1, N = 198) (see also Supplementary Fig. S1). Regarding the cytomegalovirus serology (CMV), 36.62% and 76.92% of NT and ASD mothers were immunized respectively (Chi-sq = 10.83, *p* < 0.001, df = 1, N = 97). Blood samples used for Guthrie’s test confirmed the lack of congenital hypothyroidy, mucovisidosis, drepanocytosis, phenylketonuria and congenital adrenal gland hyperplasia in both groups. They also revealed no CMV mRNA indicating that, with the limits of this test, the impact is not due to neonatal viral infection but to maternal immunization. The median of IgG CMV is 0 IU in NT babies versus 9950 IU in the ASD group (MWU = 382.5, *p* < 0.001). The strong difference of the median (0) and mean (4675.00) in the NT group reflects a right skewness of the IgG curve with at least 50% of NT babies having 0 IgG CMV levels (Fig. 3). With the FIGO classification of fetal heart rate during labour, 61.22% and 62.22% of NT and ASD babies respectively have a normal heart rate whereas 10.20% and 28.89% of NT and ASD babies respectively have a pathological heart rate (Chi-sq = 13.82, *p* = 0.001, df = 2, N = 192).

### Head circumference growth differs in NT and ASD

**Figure 4:**
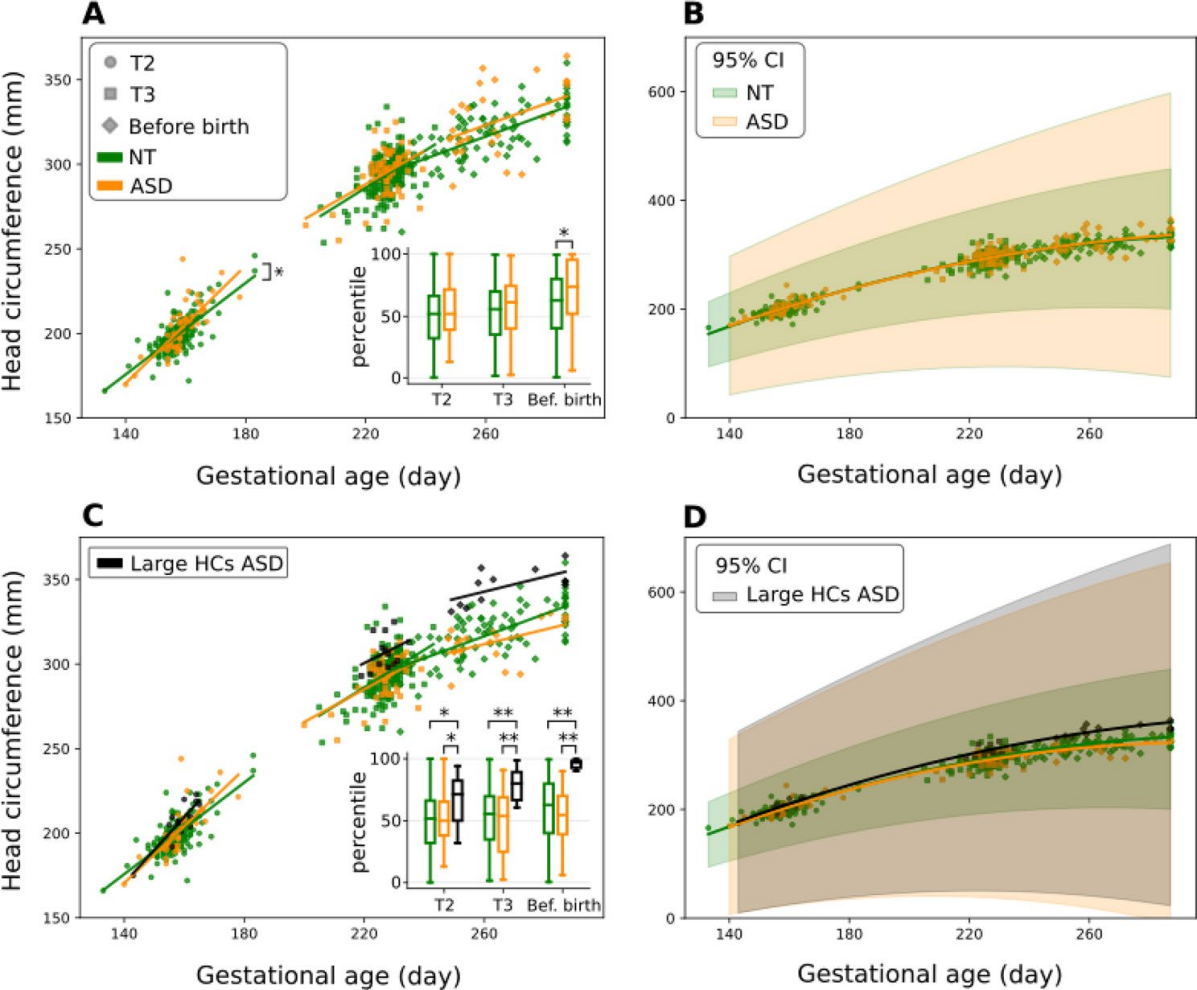
Head circumference (HC) growth is slowed down during development in NT and ASD, but shortly before birth ASD HC is bigger than NT HC. The HC in NT (green) and ASD (orange) groups is depicted versus the gestational age in T2 (circle), T3 (square) and shortly before birth (diamond) periods. (**A**) Linear regression analysis shows the growth of HC in T2 is significantly higher in ASD than NT (*p* < 0.05). Also, ASD HCs are bigger than NT shortly before birth (*p* < 0.05) as shown in the boxplot (center line, median; box limits, upper and lower quartiles; whiskers, 1.5 × interquartile range). (**B**) The quadratic mixed effect model shows a similar progressive slowdown of HC of NT and ASD children towards birth. The 95% confidence intervals are shown for each curve. (**C**) A subpopulation of the ASD group with large HCs before birth was separated from the rest of the ASD group (black points). Linear regression analysis shows similar HC growth rates in the 3 groups. However, ASD fetuses with large HCs have significantly larger HCs than NTs in the 2nd and 3rd trimester and shortly before birth (*p* < 0.05, *p* < 0.001 and *p* < 0.001, respectively). (**D**) The quadratic mixed effect model shows similar progressive slowdown of HC of NT, ASD and “Large HCs ASD” children towards birth. *p < 0.05, **p < 0.001.

The head circumference (HC) growth rate is significantly different between NT and ASD during the 2nd trimester (ANCOVA, *p* = 0.046) with the slopes of regression line equals to 1.35 for NT (*p* < 0.001, R^2^ = 0.52, Pearson’s correlation coefficient ρ = 0.72) and 1.73 for ASD (*p* < 0.001, R^2^ = 0.61, ρ = 0.78) (Fig. 4A). However, the mean percentile values of HC are not different at this age (t-test, *p* = 0.2) (box plot of Fig. 4A). During the 3rd trimester, the increasing growth slopes are similar for NT (1.11, *p* < 0.001, R^2^ = 0.23, ρ = 0.48) and for ASD (1.01, *p* < 0.001, R^2^ = 0.20, ρ = 0.45) (ANCOVA, *p* = 0.72), although there is an increasing trend in the HC percentile (MWU, *p* = 0.83). In contrast, before birth, ASD’s HC percentiles are significantly higher than NT (MWU, *p* = 0.02), but the growth slopes are similar (0.65 for NT (*p* < 0.001, R^2^ = 0.46, ρ = 0.68) and 0.63 for ASD (*p* = 0.01, R^2^ = 0.20, ρ = 0.45)) (ANCOVA, *p* = 0.93; see also Supplementary Fig. S2). The quadratic mixed effect model shows a similar (*p* = 0.30) slowdown of HC increase in the NT and ASD groups along the gestation (Fig. 4B). The coefficient of quadratic term is − 0.006 for NT (95% CI: [− 0.006, − 0.005]; *p* < 0.001) and − 0.005 for ASD (95% CI: [− 0.006, − 0.004]; *p* < 0.001). The larger CI of the ASD group suggests a larger heterogeneity in ASD than in NT. Collectively, these observations raise the possibility that brain growth is altered in ASD but might be confounded by the large heterogeneity of HCs.

Indeed, the distribution of HC percentiles reveals that 38% of ASD babies have a HC percentile higher than 90% before birth (box plot of Fig. 4A and Supplementary Fig. S2). In order to determine if this is already clearly detectable in T2 and T3, we grouped these 38% of ASD babies with large HCs at birth in a group called “Large HCs ASD” (Fig. 4C). We found that during the 2nd trimester, there is a significant difference in HC percentile between groups (ANOVA, *p* = 0.02) with babies in the “Large HCs ASD” group having bigger HCs than both NT (Tukey, *p* = 0.01) and the remaining ASD subpopulation (Tukey, *p* = 0.04) (boxplot of Fig. 4C). The significant difference is also observed during the 3rd trimester and before birth (Kruskal–Wallis, *p* < 0.001), “Large HCs ASD” have larger HCs than both NT and the remaining ASD subpopulation (Dunn, *p* < 0.001 for both groups).

Linear regression analysis with the ANCOVA test shows no difference in the HC growth rate between the 3 groups in either period (Fig. 4C). The decline in head growth rate during gestation was confirmed in “Large HCs ASD”, like for the other groups, with the quadratic mixed effect model (Fig. 4D) with a coefficient of quadratic terms of − 0.006 in ASD (95% CI: [− 0.008, − 0.005]; *p* < 0.001) and − 0.005 in “Large HCs ASD” (95% CI: [− 0.006, − 0.004]; *p* < 0.001).

Therefore, a subpopulation of babies with ASD have large HCs in the 2nd and 3rd trimesters and before birth. However, NT and all ASD babies, including the subpopulation with large HCs, share a similar slowdown of HC growth. Thus, the strong growth rate and its attenuation in preparation for birth is preserved in ASD and NT brains.

## Discussion

The difficulty of developing an early diagnosis of ASD stems from the prenatal and early postnatal origin of the disease and the heterogeneity of symptoms, which may not emerge or be identifiable early in life. Detection in toddlers before clinical signs are conspicuous is essential as early behavioral treatment ameliorates ASD deficits and attenuates long-term outcomes^32,33^.

Several attempts have been made to detect ASD early relying on neuroimaging techniques, EEG measures or genetic variants. In these studies, the prediction is centered primarily on siblings of children diagnosed with ASD, that is, high-risk populations. They are therefore hampered by this factor, as the ratio of high-risk to low-risk is not representative of the general population. Neuroimaging in babies at high familial risk of autism have revealed increased brain volume that appears before ASD diagnosis^34–36^. The authors obtained a high sensitivity and accuracy of ASD prediction, but the restriction to high-risk sibling hampers and limits the generalizability of the conclusion to firstborns without siblings with ASD^37^. Similarly, EEG power spectrum analysis of at-risk siblings from three months onwards^38^ distinguishes ASD from NT children with an accuracy (true negative and positive outcome) of 91.67%. The positive predictive value is, however, that 63.93% of those diagnosed as at-risk during the first year go on to develop ASD later. Interestingly, the frontal EEG analysis at age 3–12 months most accurately discriminated the ASD group, pointing to early perinatal processes vs. later ones, and the presence of early subclinical changes that can be detected by early frontal EEG power. Furthermore, the widely used genocentric approach has not allowed establishing an early prognostic of ASD to large populations due to several limitations. Hundreds of genetic mutations and variants have been identified often with poor penetration that produces incremental risks when cumulated^39–44^. In addition, de novo variations play an important role^45^, complicating the prediction. Many non-genetic factors play an important role in ASD pathogenesis during maternity including vitamin deficiency^11^ and environmental factors (e.g. pollution, pesticides, for review^13^).

Our goal here was to determine whether it is possible to give a prognosis of ASD shortly after birth relying on imaging and biological features that are routinely collected during pregnancy and birth. We reasoned that this would both provide compelling evidence that ASD is born in the womb, and to use data normally available in maternity wards to enable an early prognostic of babies at-risk. In this aim, ML algorithms and conventional statistical hypothesis tests were employed to analyze data collected from a representative population of ASD with a global incidence (1.21%) similar to that reported in Europe and other countries^3^. ML is useful in this context, as it enables to identify features that are not statistically significant, but that converge to impact ASD identification. Moreover, ML approaches have shown recently their power in disease prognosis with applications in hepatitis prediction^46^, classification of diabetic patients^47,48^, and lung cancer screening^49^. ML also enables to determine the interactions of each gene with all the genes of the network associated with ASD^50^. They however cannot be used to differentiate NT and ASD babies.

Our results suggest that a combination of the collected features during maternity and during birth impacts the classification and prognosis of ASD, including some features that are not intuitively linked to ASD. Many of the identified features have, at this stage, no straightforward mechanistic links with ASD, except quite indirect speculative connections. The femur length percentile differences might be related to the finger and toe ratios altered in ASD because of hormonal influences^51,52^. Gestational hypoxia^53^, like pathological heart rate during labor and birth, has been associated with neurological sequels^54^. 95% of embryos have their head down at birth^55,56^, but here we show that the shift occurs earlier in ASD possibly suggesting an earlier preparation for birth. There are less than 1 °C changes in body temperature in the majority of NT children between birth and 1 day later, but bigger differences (warmer or cooler) in ASD. This suggests a difficulty in controlling body temperature that might be related to inflammatory signals^57^. Several features associated with inflammatory signals are also significantly different in ASD and NT, including maternal immunization to CMV, the average of IgG CMV units, and familial maternal history of auto-immune diseases^8^. However, other impactful parameters such as low values of white blood cell count in T3 and newborn feeding with a mix of breast and artificial milk have, to the best of our knowledge, no documented links with ASD. Nevertheless, some identified biomarkers were highly expected because of the convergence of experimental and clinical studies. Thus, MIA has been shown to be associated with ASD in epidemiological studies^25,27,58,59^ and in experimental conditions where activation of the immune system in utero leads to ASD behavioral and physiological alterations^26,60–62^.

The developmental curve of head circumference growth in utero suggests that brain growth is impacted at a very early stage. Brain growth of NT and ASD is slowed down from the 2nd trimester to birth but with important differences between them. Although the mean HC values are not different between NT and ASD, there is a significant acceleration of growth in the latter versus the former in the 2nd trimester suggesting a long-lasting impact of the pathogenic event such that the ASD group has a significantly larger HC before birth. We also identified a subpopulation of “Large HCs ASD” with significantly larger HCs than age-matched NTs during the 2nd and 3rd trimesters and before birth. Interestingly, the HC of a subpopulation (15%) of children and adolescents with ASD has been reported to be bigger than NT with “megalencephalic” features^15,16^. Therefore, brain growth process is impacted already from the 2nd trimester with a HC that continues growing during the few days that precede birth. Future studies will have to determine if the brain continues growing *during* parturition as observed in the in utero VPA rodent model of ASD.

Indeed, experimental observations are in accord with this. Hippocampal and neocortical volumes are increased in the in utero VPA rat model of ASD and hippocampal neuron size grows during parturition and birth^26,30^. In patients with ASD, the process that governs postnatal cellular maturation, like the trajectory of neuronal development, is altered in the human amygdala with a persistence of neurons endowed with immature features^63,64^. Neurons with immature features are present in the adolescent and adult human amygdala^65^ illustrating the long lasting impacts of an in utero pathogenic event. In keeping with this, the “neuroarchaeology “ concept posits that the initial insult in utero deviates developmental sequences leading to a persistence of neurons endowed with immature features^66^. These are the direct cause of the disorder generating patterns of activity that disturb behaviorally relevant oscillations. Neurons with immature properties have been observed in many pathological conditions including animal models of ASD. High intracellular chloride levels and GABA excitatory actions- universally present in developing neurons- are observed in rodent models of MIA^26^, in utero Valproate, and Fragile X^67–69^ and Rett syndromes^70^ suggesting a common global reaction to the pathogenic insult. Interestingly, administration of the NKCC1 chloride importer antagonist Bumetanide that restores GABAergic inhibition and low intracellular chloride levels also attenuates the severity of ASD^71^ and the brain volume changes observed at birth^30^. This stands in accord with the clinical trials showing an attenuation of ASD symptoms in children with ASD^72–75^ and in adolescents/adults with Tuberous Sclerosis^76^.

There are many limitations to the present study. Perhaps the biggest limitation is the time range of data collection, since the features cannot be collected at fixed dates thereby hampering their significance. This is an intrinsic limitation common to all studies on maternity. It is, however, reduced to a large extent by the collected time course of the changes notably for the HC. In other words, we transformed raw ultrasound measurements to percentiles with respect to common distributions of ultrasound measurements in France which take into account the term of acquisition. In addition, the small sample size and the number of girls limit the generalizability of the results. On the other hand, according to our results with the statistical tests, some features such as the baby’s temperature, CMV, and IgG are distinguishing but were not included in the classification process due to their missing value rates. Moreover, we deliberately preferred to minimize false positives, which restricted ASD detection rate and feature identification. Therefore, the findings should be interpreted cautiously, in the frame of limitations and preferences of this study. These results should be considered as a proof of concept for an early prognostic tool but not an early diagnosis of ASD. Future studies might help ameliorating these aspects by considering a larger population in order to cope with the heterogeneity of ASD features and including other factors such as genetic susceptibility, EEG recordings and post-natal measurements from the first year of life. A large dataset with enough samples in each ASD subclass might enable to extrapolate the methods and findings of this study into biological or symptomatic subgroups.

To conclude, our results suggest that it might be possible to establish a prognosis at birth of a subpopulation of babies who will develop ASD. The trained algorithm will require larger replications before being considered as a clinical tool for predicting ASD in large populations, as false predictions might adversely affect individuals. Yet, our results are in keeping with various lines of evidence suggesting that ASD is generated in utero^16,42^ possibly by a pathogenic in utero sequence of events that impacts cell proliferation, migration and many other essential processes^66^. The time and structural basis of the initial insult most likely underlies the heterogeneity of ASD^42,66^. If confirmed, the identification at birth of babies at risk of ASD relying on data that are routinely available in maternity wards will facilitate the use of behavioral therapeutic strategies before the end of the developmental plasticity critical period^32,33^.

## Methods

This study has been authorized by the ethical committee of the Limoges Hospital (number 96-2019-62) and the Commission Nationale d’Informatique et des Libertés (CNIL -1632017 v 0, of 21/11/2012). All experiments were performed in accordance with relevant guidelines and regulations. Informed consent was obtained from parents or legal guardians of each baby whose data was included in the study.

### Data and experiments

In 2012–2013, 5356 babies were born in the maternity Hospital of the University of Limoges in France. Two to 5 years later, 65 of these babies (1.21%) were diagnosed with ASD (DSM-5 APA 2013) and confirmed by ADI-R and the Autism Diagnostic Observation Schedule (ADOS G). Babies whose mothers were of legal age (18 years old in France) were included in the study. Moreover, the parents of the babies must speak French and be affiliated with the French social security system. The babies had to be born alive and had no abnormalities other than ASD and no abnormalities for the NT ones. Babies for whom the vast majority of the pregnancy was followed in another maternity hospital were excluded from this study. The classical follow-up from the second ultrasound (20–24 weeks of amenorrhea) was required to be included. The ASD incidence rate in our population (1.21%) is close to the reported rate in the literature^3^, which justifies our sampling approach.

Babies were included when the term of delivery was more than 30 weeks of amenorrhea, two babies with ASD were excluded due to Trisomy 21 and extreme prematurity and a microcephaly (birth at 30 weeks). The files of the 63 babies with ASD (12 girls and 51 boys) were matched with 189 neurotypical (NT) babies based on mother's age, parity and term of childbirth. About 14% of babies were born preterm (between 30 and 37 weeks) in each group. For simplification, we shall refer to babies diagnosed years later as NT or ASD as NT or ASD babies respectively. For each mother and baby, 116 features were recorded during pregnancy until 1 day after birth. The feature space consists of 77 numerical features (e.g. mother’s BMI, ultrasound measurements), 38 categorical (e.g. sex, familial medical history, auditory tests), and 1 ordinal (placenta Grannum classification in the 3rd trimester), which are commonly recorded in French maternity hospitals*.* The Supplementary Table S4 provides the entire list of features used in this study (see also Supplementary Methods).

Ultrasound measurements (femur length, head circumference, abdominal perimeter, etc.) were performed according to the recommendations of the French College of Fetal Ultrasound (http://www.cfef.org/). The measurement dates are 10–14, 20–24 and 30–34 weeks of amenorrhea. In addition, supernumerary ultrasounds were performed during pregnancy for various reasons such as hypertension, diabetes, etc.

Head rotation can be observed during an ultrasound scan or during a clinical examination. What was reported is the first mention of a passage head down from 22 weeks of amenorrhea. It is also noted whether this passage is perennial or, on the contrary, if it was only transitory.

The goal of this study was to find patterns in the recorded features that distinguish ASD babies from NT ones, and our approach to this goal was 2-folded. First, a supervised classification algorithm was trained on data, and features with high impact on the classifier were identified with two different methods. In the second approach, appropriate statistical hypothesis tests were performed to find features that have significantly different distributions in NT and ASD groups. Moreover, developmental trajectories of head circumference were studied by statistical models.

### Data preprocessing

The values of each 116 recorded features in the dataset were explored and cleaned carefully to reduce the noise in the computations. Features with a missing value rate higher than 10% were removed from the classification process to reduce the imputation bias in the results. Features that were included in the classification process are given in the Supplementary Table S5. Consequently, the classification dataset consists of 67 features for which 2.58% of values are missing in total. The one-hot encoding technique^77^ was applied to binarize categorical features. To avoid co-linearity, one category of each feature was dropped.

### Feature selection for classification

A common issue in technology-based biological classification studies is the low ratio of sample size to number of collected features^78^ which increases the classification error and the risk of data overfitting^79–81^. To treat this issue, a good practice is reducing the dimension of the feature space by finding and dropping irrelevant and redundant features based on some criteria or domain knowledge.

In this study, features were selected by two strategies: the automatic strategy by the Lasso regularization technique^82^, and semi-automatic strategy which consists of a feature preselection based on medical knowledge followed by the Lasso technique. The goal was to compare classification performance with and without human intervention in feature selection and also, the similarity between selected features by those strategies.

The automatic strategy relies on a Lasso regularization technique that is applied directly in the training process of the classifier. It shrinks the impact of irrelevant features on the classification and selects implicitly the most distinguishing ones. This technique is known to be very effective even in the presence of many very irrelevant features^82^, and may find some features that are not already known to be linked to ASD. The semi-automatic strategy investigated the effect of feature preselection by using domain knowledge before applying the Lasso technique. In this strategy, 19 features (out of 67) that might be linked to ASD were preselected by 3 experienced obstetricians and fed to the training process (Supplementary Table S5). Among those features, the most informative ones were selected by the Lasso technique.

### Classification process

To classify babies as NT or ASD, we used a model based on the gradient boosting decision tree algorithm^83^. This is a nonparametric supervised learning method which uses a tree-like model to infer a decision for each baby from feature values. Instead of using only one tree model, an ensemble of them is considered under the gradient boosting technique to fortify the ability of the classifier. Starting with a simple classification tree model, the model learns by adding more trees in an iterative manner to minimize a learning objective. It can detect complex underlying patterns of features to predict the binary target variable of belonging to the ASD group. This algorithm gives state-of-the-art results in a wide range of classification applications, especially in healthcare and diagnosis of diseases^46,47,84,85^.

To implement the gradient boosting decision tree algorithm efficiently, we relied on the eXtreme Gradient Boosting (XGBoost) library^86^. Tuning its hyper-parameters to control the implementation of the algorithm enabled to resolve many classification problems (see https://github.com/dmlc/xgboost/blob/master/demo/README.md). Moreover, XGBoost has a built-in strategy to deal with missing values by finding the best imputation^86^.

In this study, we used a nested cross-validation process to tune the number of decision trees and evaluate the classifier’s performance (see below). Moreover, we tuned carefully several hyperparameters to control the complexity of the model and avoid overfitting. Namely, the depth of each tree was set to a relatively low value as 5. The model weights were shrunk after each learning iteration by a factor of 0.01. The features were subsampled in each tree to make the model robust to potential noise in the data. The rate of subsampling was inversely proportional to the number of features and was selected as 0.7 and 0.5 in semi-automatic and automatic feature selection strategies (FSS) respectively. We used Lasso and Ridge regularization techniques to impose a penalty on the complexity of the classifier. Lasso regularization, as explained above, also helped to detect and remove less relevant features automatically which, in turn, avoided overfitting.

Imbalanced datasets are common in medical studies due to the low prevalence of diseases. This causes a classifier to learn mostly patterns in the majority class, i.e. control samples. To cope with this issue, we imposed a higher weight on the misclassification error of ASD samples than that of NT ones. The classifier output for each baby is the probability of the baby to belong to the ASD group. We set the decision threshold to 0.5 to binarize the predicted probability.

By choosing higher weights on ASD misclassification error or lower values of the decision threshold than those considered here, more ASD babies could be detected, but false positive rate would increase as well, which is in contrast to our ethical concerns and would increase the risk of overfitting. The list of XGBoost hyperparameters with corresponding values used in this study is given in Table 4.

**Table 4:** Hyperparameters of the XGBoost classifier.

Hyperparameter	Values	Hyperparameter	Values
Number of trees	10, 20, 30	Feature subsampling rate	0.7 or 0.5
Tree depth	5	ASD sample weight	2
Learning rate	0.01	Lasso and ridge coefficients	10

The feature subsampling rate is set to 0.7 and 0.5 for semi-automatic and automatic feature selection strategies respectively.

### Hyperparameter tuning and classifier evaluation

To tune the hyperparameters of the XGBoost classifier and to evaluate its performance, we used a 10-times repeated nested tenfold stratified cross-validation (CV) process. In each repetition of the CV process, the whole dataset was divided randomly in 10 partitions, 9 partitions for training the classifier and 1 held-out partition to test the trained classifier and to ensure that the algorithm can be generalized in future unseen samples. The train-test process of the classifier ran in 10 rounds. In each round, the hyperparameters of the classifier were tuned on train data through an internal five-fold stratified cross-validation grid search on values given in the Table 4. Optimal hyperparameters were chosen to maximize the *F*_*0.5*_ score of the classification (see below for the definition of the *F*_*0.5*_ score). The model was trained using optimal hyperparameters on the train data and the trained model was used to predict the target variable of samples in the 1 held-out test partition. Beyond *F*_*0.5*_, local classification scores, including True Positive Rate (TPR, aka sensitivity), True Negative Rate (TNR, aka specificity) and Positive Predictive Value (PPV, aka precision) were recorded at the end of each round. This procedure was repeated 10 times with different random partitionings of the dataset resulting in 100 rounds of train-test processes. Finally, the averages of the recorded classification scores were considered as the final cross-validated scores of the classifier.

The 10-times repetition of the CV procedure reduces the effect of bias on classification scores due to a relatively small number of samples. It ensures that the classification scores and hyperparameter tuning are not affected by any specific train-test partitioning of the dataset and the classifier is generalizable to future unseen samples.

### F_0.5_ score

Regarding the ethical aspects of this study, we decided to minimize false positives, in the first place, while detecting ASD samples as much as possible. This goal could be achieved by maximization of PPV but it would be at a cost of decreasing TPR. To treat this issue, we chose to maximize the *F*_*0.5*_ score which balances the PPV and the TPR while it puts higher weight on PPV, i.e. it pays more attention on minimization of false positives:

$${F}_{0.5}=\left(1+{0.5}^{2}\right)\times \frac{\mathrm{PPV} \times \mathrm{TPR}}{\left({0.5}^{2}\times \mathrm{PPV}\right)+\mathrm{TPR}}.$$

### Important feature identification

An important goal of ML analysis in this study was to identify features that separate effectively NT from ASD babies. Our approach to this goal was 2-folded: (1) finding features that appear more frequently in the CV process; (2) finding features with the highest impact on the classifier’s decisions. In the first approach, selected features by the classifier in each CV fold were recorded. At the end of the CV process, the frequency of each feature was computed. Those features that appeared more frequently in the CV process were more important for classification.

In the second approach, the classifier was trained by all 252 samples and the classifier’s output was explained by the novel SHapley Additive exPlanations (SHAP) framework^87^. This method works in the level of each sample and feature, and it provides more details than the first approach. It provides SHAP values ${s}_{ij}$ that indicates the impact of feature _*j*_ on the classifier’s decision for baby *i*. A positive or negative ${s}_{ij}$ means that feature *j* pushes the classifier to classify the baby *i* in the ASD or NT group, respectively. The higher the absolute value $\left|{s}_{ij}\right|$, the bigger impact of feature *j* on the classifier’s decision for baby *i*. On the other hand, ${s}_{ij}\approx 0$ implies a very low impact.

The total impact of all features for all 252 babies is computed as:

$$\mathrm{Total~impact~of~all~features}={S}_{T}=\sum_{i=1}^{252}\sum_{j=1}^{{n}_{f}}\left|{s}_{ij}\right|,$$

where *n*_*f*_ is the number of selected features by the FSS. The absolute impact of each feature is calculated as:

$$\mathrm{Absolute~impact~of~feature~} j={S}_{j}=\sum_{i=1}^{252}\left|{s}_{ij}\right|,~~ j=\mathrm{1,2},\dots , {n}_{f}.$$

The relative impact of each feature in percent is given by:

$$\mathrm{Relative~impact~of~feature~} j=\frac{{S}_{j}}{{S}_{T}}\times 100,~~ j=\mathrm{1,2},\dots , {n}_{f}.$$

The relative impact is used to rank the features and to identify the most impactful ones.

### Feature correlation

It is very common to observe a correlation between biological features. In one hand, correlated features are known to ruin the stability of ML algorithms. On the other hand, it is not obvious a priori which ones are more predictive to keep in the classification process. To cope with this situation, we deliberately chose to work with the XGBoost algorithm since decision tree-based models are by nature robust to correlated features. Moreover, in boosting models, in contrast to bagging models (e.g. Random Forests), the feature importance is not diluted between correlated features. All the importance is assigned to one of the correlated features. However, the feature that is chosen by the algorithm may be less biologically relevant than its correlated counterparts.

Therefore, we computed the correlation coefficients between all the features that are present in the classification process in order to find the ones which are highly correlated with identified biomarkers. For this analysis, we computed the Spearman’s correlation coefficient between numerical features, Chi-squared test of independence and Cramér’s V measure of association between categorical features, and the correlation ratio between categorical and numerical features.

### Statistical analysis

The difference in the distribution of 116 collected features between NT and ASD groups were investigated by using conventional statistical hypothesis tests. For categorical features, the Chi-squared test (Chi-sq) was used. The two-sided Welch’s t-test (t-test with Welch correction for variance heterogeneity) was applied on numerical parameters when the normality assumption was plausible according to the Shapiro–Wilk normality test. Otherwise, the nonparametric two-sided Mann–Whitney U test (MWU) was used with the Levene’s test of variance homogeneity. Moreover, the Benjamini–Hochberg procedure was employed to decrease the false discovery rate which adjusted the significance level to α = 0.001.

We used Analysis of covariance (ANCOVA) to model the fetal brain developmental trajectories, measured as head circumference (HC) from ultrasound acquisitions during the 2nd and 3rd trimesters and before birth. It also lets us test if the growth rate (slope of regression line) changes between NT and ASD at each age (level of significance α = 0.05). Inspired by the collective HC distribution from the 2nd trimester to before birth, a quadratic mixed effect model was fitted to determine the brain growth rate in this period. The interaction term between ultrasound acquisition day and ASD or NT condition was considered as a fixed effect. Random intercepts and slopes were included to take inter-individual baseline and growth rate variabilities into account.

Distributions of ASD HC percentile before birth revealed a negative skewness where about 38% of ASD babies had a large HC percentile (> 90%). We conjectured that those fetuses have already had large HC in the 2nd or 3rd trimester. To examine that, those fetuses were separated from the other ASD samples and they formed the “Large HCs ASD” group. The difference in HC percentile distributions of the 3 groups i.e. NT, ASD and “Large HCs ASD”, was checked by ANOVA with Tukey’s post-hoc test (when the normality assumption and homogeneity of variance were plausible) or the Kruskal–Wallis test with Dunn’s post-hoc test in the 2nd and 3rd trimesters and before birth. Moreover, the brain growth of this group was compared to that of other ASD and NT babies by using ANCOVA and a quadratic mixed effect model as described before.

### Implementation

All the programming and implementation of XGBoost was done on Python v.3.6 using the NumPy v.1.18.1, Pandas v.0.24.2, scikit-learn v.0.22.1, Matplotlib v.3.0.2 and XGBoost v.0.80 libraries. The impact of the parameters was calculated thanks to the SHAP library v.0.28.5. Moreover, we used the SciPy v.1.1.0 and StatsModels v.0.10.0 libraries for statistical tests, linear regression analysis and mixed effect analysis.

## Supplementary Information

Supplementary Figures.

Supplementary Methods.

Supplementary Table S1.

Supplementary Table S2.

Supplementary Table S3.

Supplementary Table S4.

Supplementary Table S5.

## Data Availability

The data and the computer codes that support the findings of this study are available from BABiomedical company, but restrictions apply to the availability of these data, which were used under license for the current study, and so are not publicly available. The data and codes are however available from the corresponding author upon reasonable request and with permission of BABiomedical company. Moreover, all processes have been described in detail to enable independent replication of results.
