# Supplementary material for: Machine learning analysis of pregnancy data enables early identification of a subpopulation of newborns with ASD
Source: Sci Rep. 2021 Mar 25;11:6877. doi: 10.1038/s41598-021-86320-0 (PMC7994821; doi:10.1038/s41598-021-86320-0)
Supplement: Supplementary file 1 — Supplementary Figures. [file 41598_2021_86320_MOESM1_ESM.docx]

# **Supplementary Figures**

**Machine learning analysis of pregnancy data enables early identification of a subpopulation of newborns with ASD**

Hugues Caly ^1+^, Hamed Rabiei ^2,3+^, Perrine Coste-Mazeau ^1^, Sebastien Hantz ^4,5^, Sophie Alain ^4,5^, Jean-Luc Eyraud ^1^, Thierry Chianea ^6^, Catherine Caly ^1^, David Makowski ^7^, Nouchine Hadjikhani ^8,9^, Eric Lemonnier ^10^, Yehezkel Ben-Ari ^2,3*^

1. Gynecology-Obstetrics Department, Mère-Enfant Hospital, University Hospital Center, Limoges, France

2. BABiomedical, Luminy Scientific Campus, Marseille, France

3. Neurochlore, Luminy Scientific Campus, Marseille, France

4. Bacteriology-Virology-Hygiene Department, University Hospital Center, Limoges, France

5. French National Reference Center for Herpes Viruses, University Hospital Center, Limoges, France

6. Department of Biochemistry and Molecular Genetics, Dupuytren University Hospital, Limoges, France

7. INRAE, UMR MIA 518 INRA AgroParisTech Université Paris-Saclay, Paris, France

8. Martinos Center for Biomedical Imaging, Harvard Medical School, Boston, USA

9. Gillberg Neuropsychiatry Center, Sahlgrenska Academy, Gothenburg University, Sweden

10. Autism Expert Center and Autism Resource Center of Limousin, University Hospital Center, Limoges, France

+ Equally contributing authors

* Corresponding author

**Address for correspondence**

Yehezkel Ben-Ari

Neurochlore

Bâtiment Beret Delaage, Case 922

Zone Luminy Entreprises Biotech, 163 Avenue de Luminy, 13288 Marseille Cedex 09, France

[ben-ari@neurochlore.fr](mailto:ben-ari@neurochlore.fr)

**
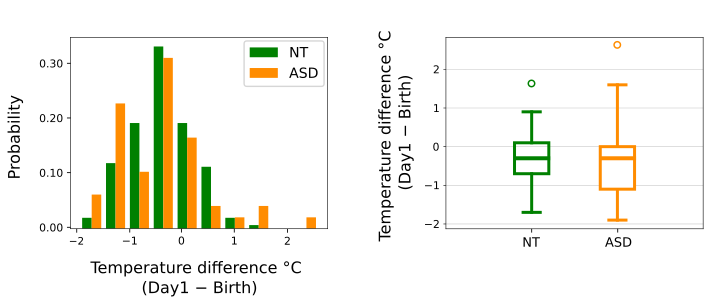
**

**Figure S1.** Left**:** Distribution of child’s temperature difference between day 1 and birth in the Neurotypical (NT; green) and ASD (orange) groups. Right**:** Heterogeneity of temperature difference in the ASD group leads to a significant difference between the NT and ASD groups in two tails of the distributions such that the number of children with temperature difference more than 1°C is significantly larger in the ASD than in the NT group. For quantitative information, see Table 3.

**
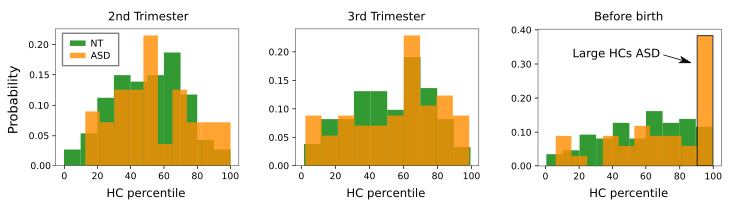
**

**Figure S2.** Distribution of head circumference (HC) percentiles in the NT (green) and ASD (orange) groups. During each 2^nd^ and 3^rd^ trimesters, the distribution of HC percentiles is quite similar in both groups. In contrast, there is a subpopulation of the ASD group with obviously large HC shortly before birth. We call it “Large HCs ASD” and analyzed them separately (see Fig. 4c and 4d).
