# Supplementary material for: Machine learning analysis of pregnancy data enables early identification of a subpopulation of newborns with ASD
Source: Sci Rep. 2021 Mar 25;11:6877. doi: 10.1038/s41598-021-86320-0 (PMC7994821; doi:10.1038/s41598-021-86320-0)
Supplement: Supplementary file 2 — Supplementary Methods. [file 41598_2021_86320_MOESM2_ESM.docx]

# **Supplementary Methods**

**Machine learning analysis of pregnancy data enables early identification of a subpopulation of newborns with ASD**

Hugues Caly ^1+^, Hamed Rabiei ^2,3+^, Perrine Coste-Mazeau ^1^, Sebastien Hantz ^4,5^, Sophie Alain ^4,5^, Jean-Luc Eyraud ^1^, Thierry Chianea ^6^, Catherine Caly ^1^, David Makowski ^7^, Nouchine Hadjikhani ^8,9^, Eric Lemonnier ^10^, Yehezkel Ben-Ari ^2,3*^

1. Gynecology-Obstetrics Department, Mère-Enfant Hospital, University Hospital Center, Limoges, France

2. BABiomedical, Luminy Scientific Campus, Marseille, France

3. Neurochlore, Luminy Scientific Campus, Marseille, France

4. Bacteriology-Virology-Hygiene Department, University Hospital Center, Limoges, France

5. French National Reference Center for Herpes Viruses, University Hospital Center, Limoges, France

6. Department of Biochemistry and Molecular Genetics, Dupuytren University Hospital, Limoges, France

7. INRAE, UMR MIA 518 INRA AgroParisTech Université Paris-Saclay, Paris, France

8. Martinos Center for Biomedical Imaging, Harvard Medical School, Boston, USA

9. Gillberg Neuropsychiatry Center, Sahlgrenska Academy, Gothenburg University, Sweden

10. Autism Expert Center and Autism Resource Center of Limousin, University Hospital Center, Limoges, France

+ Equally contributing authors

* Corresponding author

**Address for correspondence**

Yehezkel Ben-Ari

Neurochlore

Bâtiment Beret Delaage, Case 922

Zone Luminy Entreprises Biotech, 163 Avenue de Luminy, 13288 Marseille Cedex 09, France

[ben-ari@neurochlore.fr](mailto:ben-ari@neurochlore.fr)

1. **Pregnancy and delivery follow-up**
2. **Pregnancy follow-up**:

Pregnancy follow-up was carried out in a standardized manner according to the recommendations of the Collège National des Gynécologues Obstétriciens Français ([www.cngof.fr](http://www.cngof.fr)) through a reference document published by the AUDIPOG association ([www.audipog.net](http://www.audipog.net)). This monitoring is based on three axes:

- A monthly consultation from the end of the first trimester to the end of pregnancy.

- Any pregnancy follow-up should include at least three ultrasounds at 12 (between 11 and 13 weeks and 6 days), 22 (20-25) and 32 (30-35) weeks of amenorrhea with the patient's informed consent.

- The third concerns biological monitoring with different controls depending on the patients and the problems encountered.

1. **Consultations:**
2. Classic follow-up:

During this consultation, in addition to urine analysis (glycosuria, hematuria, acetonuria, nitrituria, proteinuria and leukocyturia), weight gain and blood pressure measurement, an anamnesis is performed with search for prescribed treatment and complications such as metrorrhagia or other. A non-systematic clinical examination is also performed with fetal position, length of cervix and an ultrasound may be performed if there is any doubt as to presentation or abnormality.

1. Supernumeraries:

Any problem can lead to a supernumerary consultation, such as an acute urinary tract infection. Similarly, a detected pathology may lead to the implementation of stricter monitoring with closer clinical surveillance.

1. **Ultrasound scans:**
2. Classic follow-up:
3. First trimester ultrasound:

This ultrasound is done between 11 and 13 weeks of amenorrhea and 6 days (i.e. a craniocaudal length between 45 and 84 mm). After information and signature of a consent form, this examination is performed by a doctor or midwife sonographer. This sonographer must meet different validation criteria:

- Adequate training and diploma.
- Join a perinatal network associated with a Multidisciplinary Centre for Pre-Natal Diagnosis.
- Adhere to a quality assurance program for nuchal transluciency and craniocaudal length measurements as part of the professional practical evaluation and produce images to document the quality of the measurements made (BioNuQual depending on the Biomedicine Agency: <http://www.bionuqual.org>)
- Monitor its medians and the distribution of its measurements of nuchal transluciency.

This ultrasound includes various mandatory measures:

o Cranio-caudal length.

o Biparietal diameter.

o Femur length.

o Nuchal transluciency.

o Validation of the quality of the ultrasound by a Herman score which must be higher than 4.

1. Ultrasound of the second trimester:

This ultrasound is done between 20 and 24 weeks of amenorrhea. It is subject to the same quality constraints as the first trimester ultrasound. It must make an exhaustive morphological approach with essential parameters.

o Biparietal diameter and head circumference (measurement and percentile).

o Abdominal diameter and abdominal perimeter (measurement and percentile).

o Femoral length (measurement and percentile).

o Cerebellum (measure and percentile).

o Lateral ventricles (measurement and percentile).

o Bones of the nose (measurement).

o Foot bones (measurement).

o Estimation of fetal weight (estimation and percentile).

1. Third trimester ultrasound scan:

This ultrasound is done between 30 and 34 weeks of amenorrhea. It is subject to the same quality constraints as the second trimester ultrasound.

o Biparietal diameter and head circumference (measurement and percentile).

o Abdominal diameter and abdominal perimeter (measurement and percentile).

o Femoral length (measurement and percentile).

o Transverse diameter of the cerebellum (measurement and percentile).

o Lateral ventricles (measurement and percentile).

o Bones of the nose (measurement).

o Foot bones bones (measurement).

o Estimation of fetal weight (estimation and percentile).

Ultrasounds estimate measurements that are established according to abacuses:

- - Neck clarity: Fetal nuchal translucency: ultrasound screening for fetal trisomy in the first trimester of pregnancy (*64*).
  - Cranio-caudal length.
  - Fetal biometries (head circumference, biparietal diameter, femoral length) have been defined in their percentile according to the formula recommended by the Collège National des Gynécologues Obstétriciens Français on reference from the Collège Français d'ultrasographie Fœtale (*65*).
  - The estimate of fetal weight (*66*).
  - The diameter of the cerebellum (*67*).
  - The diameter of the lateral ventricle (*68*).

1. Supernumeraries:

There are many reasons to perform an additional ultrasound to those recommended. These may include position control, and consultation reasons requiring control such as: decreased fetal active movement, suspicion of hydramnios or premature rupture of membranes.

1. Concerning the position of the fetal head:

A major item in this study is the time of rotation of the fetus upside down. Knowing that this item was at the very least unexpected, we wanted to check its validity and we looked for this position during the first consultation mentioning this nutation. To validate this fact, we recovered this information during the second trimester ultrasound and during the additional ultrasound if it existed in order to have strong indications of the reality of this rotation. In each case, it appeared that children with ASD turned faster than NT.

1. **Biological analysis:**
2. Serology:

The recommended serologies during pregnancy are:

- Toxoplasmosis to be renewed monthly if negative.
- Rubella to check vaccination status.
- Hepatitis B to verify vaccination status.
- Hepatitis C.
- Syphilis.
- HIV.
- Optional cytomegalovirus (CMV) and is only prescribed under certain circumstances (e. g. hydramnios).

1. Diabetes screening:

It includes fasting blood glucose in the first trimester and a sugar test at 26 weeks of amenorrhea with 75 grams of sugar ingested.

1. Blood type, Rhesus and search for irregular agglutinins.

IV. Serum trisomy 21 (T21) risk markers:

They include a blood test with two markers in the first trimester, namely free HCG and PAPP-A. These peptides are measured, and their gross value is given in DoE (division of extreme) or MoM (multiple of the average) depending on the laboratory concerned. During the period in question, it was a german laboratory (https://www.brahms.de) rendering their results in the form of DoE, which is not the most frequent format but does not affect the rendering.

The result includes an integration of these dosages which are weighted with different factors such as the patient's age, origin, maternal tobacco use, nuchal translucency measurement, etc. and it results in a risk estimate that must be less than 1/250. However, the rendering of these results limits the denominator to 10,000. We have taken into account the real number of the denominator which is much more informative than an arbitrary limit.

There are other forms of calculating the risk of T21 in the second trimester with other serum markers. For the sake of consistency, we have only retained files that take into account first trimester markers.

V. End of pregnancy check-up:

- Blood count.
- Coagulation assessment.
- Vaginal sampling for streptococcus B.

1. **Delivery :**
   1. **Maternal supervision:**

Birth monitoring begins with the mode of labor (spontaneous, induction, scheduled or emergency Caesarean section), the type of analgesia performed, and the treatments used for induction and subsequent labor (antibiotic, oxytocin, etc.). Maternal hourly monitoring includes blood pressure, pulse rate, degree of analgesia and body temperature. The duration of each phase of labor, rupture of membranes and epidural analgesia were reported.

- 1. **Fetal surpervision:**

Fetal monitoring includes continuous cardiac monitoring throughout the duration of labor until the child is expelled, coupled with a uterine contraction recorder. The fetal heart rate of each patient was read by three different obstetricians not knowing which group the patient belonged to. It has been evaluated according to the 2015 FIGO classification whose rules are described in the table below ^1^.

**
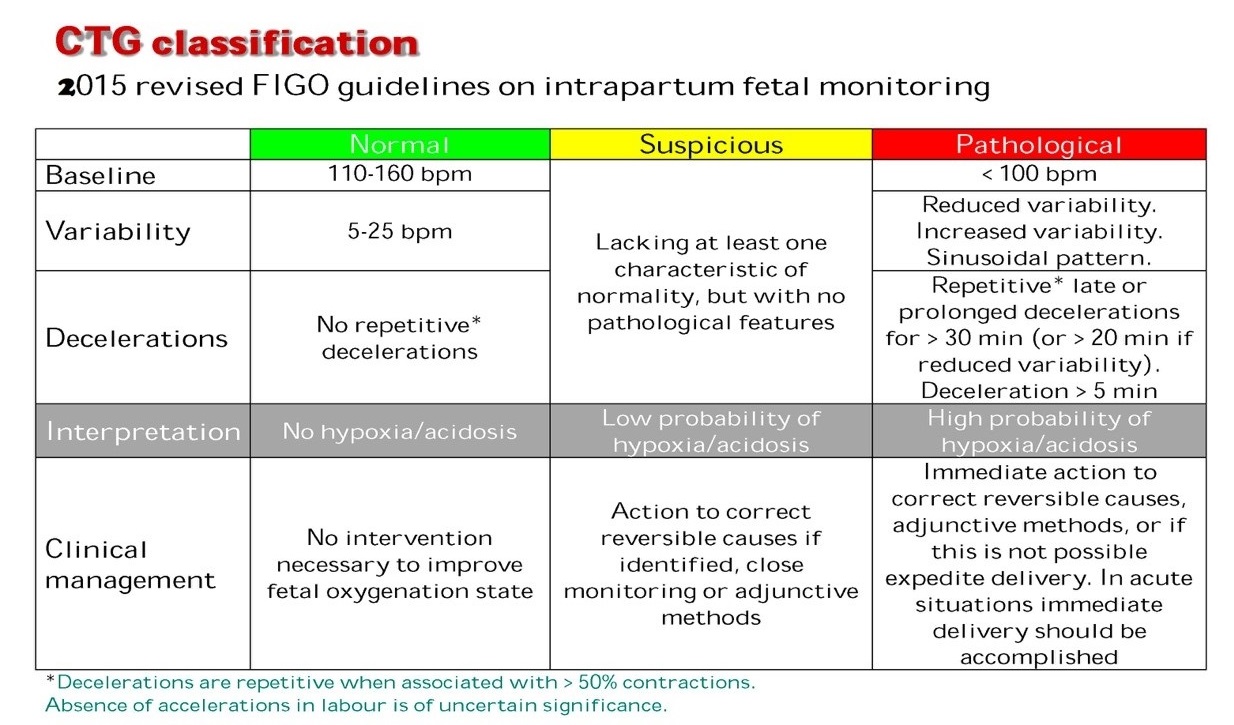
**

- 1. **Newborn surveillance:**

1. At birth:

This supervision includes a care in order to ensure the well-being of the child according to various controls:

- Apgar’s Score at 1, 3, 5 10 minutes.
- Temperature.
- Arterial pH with cord blood, pCO2 or lactates.
- Weight, weight percentile according to AUDIPOG curves ([www.audipog.net](http://www.audipog.net)).
- Skin to skin protocol.
- Type of breastfeeding.

1. First day of life:

Every day during the maternity stay, the child is weighed, and his/her temperature is taken. A complete pediatric examination is done the day after birth and is repeated on average two days later. The head circumference and size are measured and recorded in percentiles according to the AUDIPOG curves ([www.audipog.net](http://www.audipog.net)). The occurrence of the first urine and meconium is noted. An examination of external hearing potentials is systematically carried out most often on the second day of life and repeated if the first one is not normal.

All this data is compiled in a computer file based on the File Maker Pro software developed by Claris International Inc. This file has been certified by the "Commission Nationale Informatique et Libertés" (1632017 v 0, of the 21/11/2012).

1. **CMV**

Parents of children with ASD were asked to participate in the study and gave their non opposition to use the dried blood spot (DBS or Guthrie card) collected at birth and stored in the regional screening laboratory.

1. DNA extraction from DBS: After cutting the DBS and lysis with sodium hydroxide, the paper was eliminated, and the lysate was transferred into the SaMag extractor for total DNA extraction with the SaMag DNA Blood Extraction Kit.
2. Albumin DNA amplification: To control the quality of the extraction, an in-house developed qPCR targeting the albumin gene was performed on each sample.
3. CMV DNA amplification: Amplification of CMV DNA was performed using the R-GENE CMV kit (bioMérieux) according to the manufacturer’s instructions. All extracts were amplified in duplicate.
4. Maternal CMV serology: The cytomegalovirus IgG detection and quantification was performed in the virology laboratory of Limoges University Hospital by the enzyme immunoassay Enzygnost CMV IgG (Siemens healthcare diagnostics). Results are expressed in UI/mL.

**References:**

1. Ayres-De-Campos, D., Spong, C. Y. & Chandraharan, E. FIGO consensus guidelines on intrapartum fetal monitoring: Cardiotocography. *Int. J. Gynecol. Obstet.* **131**, 13–24 (2015).
