# Supplementary material for: Machine learning analysis of pregnancy data enables early identification of a subpopulation of newborns with ASD
Source: Sci Rep. 2021 Mar 25;11:6877. doi: 10.1038/s41598-021-86320-0 (PMC7994821; doi:10.1038/s41598-021-86320-0)
Supplement: Supplementary file 5 — Supplementary Table S3. [file 41598_2021_86320_MOESM5_ESM.docx]

# **Supplementary Table S3**

**Machine learning analysis of pregnancy data enables early identification of a subpopulation of newborns with ASD**

Hugues Caly ^1+^, Hamed Rabiei ^2,3+^, Perrine Coste-Mazeau ^1^, Sebastien Hantz ^4,5^, Sophie Alain ^4,5^, Jean-Luc Eyraud ^1^, Thierry Chianea ^6^, Catherine Caly ^1^, David Makowski ^7^, Nouchine Hadjikhani ^8,9^, Eric Lemonnier ^10^, Yehezkel Ben-Ari ^2,3*^

1. Gynecology-Obstetrics Department, Mère-Enfant Hospital, University Hospital Center, Limoges, France

2. BABiomedical, Luminy Scientific Campus, Marseille, France

3. Neurochlore, Luminy Scientific Campus, Marseille, France

4. Bacteriology-Virology-Hygiene Department, University Hospital Center, Limoges, France

5. French National Reference Center for Herpes Viruses, University Hospital Center, Limoges, France

6. Department of Biochemistry and Molecular Genetics, Dupuytren University Hospital, Limoges, France

7. INRAE, UMR MIA 518 INRA AgroParisTech Université Paris-Saclay, Paris, France

8. Martinos Center for Biomedical Imaging, Harvard Medical School, Boston, USA

9. Gillberg Neuropsychiatry Center, Sahlgrenska Academy, Gothenburg University, Sweden

10. Autism Expert Center and Autism Resource Center of Limousin, University Hospital Center, Limoges, France

+ Equally contributing authors

* Corresponding author

**Address for correspondence**

Yehezkel Ben-Ari

Neurochlore

Bâtiment Beret Delaage, Case 922

Zone Luminy Entreprises Biotech, 163 Avenue de Luminy, 13288 Marseille Cedex 09, France

[ben-ari@neurochlore.fr](mailto:ben-ari@neurochlore.fr)

**Table S3.** For each feature, statistical information of sample distributions in ASD and neurotypical (NT) groups are given. In case of categorical features, frequency (%) and number (n) of samples in each category are shown. In the case of numerical features, median, mean, standard error of mean, 95% confidence interval of mean are given respectively. Results of the statistical tests between ASD and NT groups are presented. Chi-sq, Chi-squared test. T-test, Welch’s t-test. MWU, Mann-Whitney U test.

| Feature | | Statistics | | | |
| --- | --- | --- | --- | --- | --- |
|  |  | ASD group | NT group | Stat. test | p-value |
| Sex | Male % (n) | 80.95 (51) | 48.70 (92) | Chi-sq | <0.001 |
|  | Female % (n) | 19.05 (12) | 51.30 (97) |  |  |
|  | Ratio M/F | 4.25 | 0.95 |  |  |
| Timing of fetal rotation on head (days) | | 166 (178.35, 4.81, 168.72-187.98) | 180 (186.55, 2.30, 182.01-191.09) | MWU | 0.03 |
| Child’s temperature difference  day 1 − birth | > 1° % (n) | 41.66 (20) | 14.00 (21) | Chi-sq | <0.001 |
|  | < 1° % (n) | 58.33 (28) | 86.00 (129) |  |  |
| Nasal bone length in T2 | | 7.50 (7.16, 0.20, 6.76-7.56) | 7.75 (7.68, 0.07, 7.55-7.81) | T-test | 0.017 |
| Foot length in T2 | | 41.00 (41.28, 0.55, 40.14-42.42) | 40.25 (40.26, 0.21, 39.84-40.69) | MWU | 0.10 |
| Femur length percentile in T3 | | 45.62 (45.60, 3.61, 38.36-52.84) | 38.20 (39.19, 1.63, 35.97-42.42) | MWU | 0.13 |
| Nasal bone length in T3 | | 11.00 (10.89, 0.20, 10.47-11.31) | 11.50(11.13, 0.11, 10.92-11.35) | MWU | 0.13 |
| Abdominal perimeter percentile in T2 | | 58.91 (59.02, 2.82, 53.36-64.68) | 55.38 (56.18, 1.67, 52.89-59.48) | MWU | 0.40 |
| Head circumference percentile in T2 | | 51.60 (54.61, 3.09, 48.42-60.79) | 51.60 (50.15, 1.54, 47.12-53.18) | T-test | 0.20 |
| Familial maternal history of auto-immune diseases | Yes % (n) | 19.05 (12) | 6.35 (12) | Chi-sq | 0.0064 |
|  | No % (n) | 80.95 (51) | 93.65 (177) |  |  |
| White cells in T3 | | 9800.00 (10039.84, 403.10, 9233.52-10846.15) | 10230.00 (10494.00, 204.56, 10090.17-10897.83) | MWU | 0.12 |
| Real first trimester markers | | 4500.00 (10320.49, 2425.53, 5438.15-15202.84) | 7901.00 (15034.73, 2456.60, 10183.42-19886.05) | MWU | 0.11 |
| Head circumference percentile before birth | | 73.58 (69.03, 5.04, 58.78-79.28) | 62.56 (58.53, 2.74, 53.07-63.98) | MWU | 0.021 |
| pCO2 on arterial blood at the umbilical cord | | 56.05 (54.54, 1.60, 51.28-57.79) | 50.85 (50.43, 0.70, 49.05-51.81) | MWU | 0.028 |
| CMV | Immunized % (n) | 76.92 (20) | 36.62 (26) | Chi-sq | <0.001 |
|  | Negative % (n) | 23.08 (6) | 63.38 (45) |  |  |
| Delta weight percentile between birth and day 1 | | 7.46 (10.51, 1.35, 7.80-13.23) | 6.79 (9.51, 0.68, 8.16-10.86) | MWU | 0.71 |
| Nucal transluciency percentile in T1 | | 25.00 (31.80, 3.93, 23.88-39.72) | 30.00 (36.63, 2.04, 32.62-40.65) | MWU | 0.34 |
| Fetal weight estimation percentile in T2 | | 55.75 (56.87, 2.87, 51.12-62.62) | 51.60 (51.36, 1.86, 47.69-55.03) | MWU | 0.18 |
| Lateral ventricle size percentile in T2 | | 5.00 (22.36, 5.58, 10.84-33.88) | 5.00 (16.53, 1.69, 13.19-19.87) | MWU | 0.78 |
| Duration of the first part of the labour (minute) | | 28.00 (249.74, 27.05, 195.65-303.84) | 267.00 (262.53, 15.00, 232.95-292.12) | MWU | 0.52 |
| Child's temperature at day 1 | | 36.70 (36.74, 0.05, 36.64-36.84) | 36.90 (36.91, 0.03, 36.85-36.96) | MWU | 0.0030 |
| Transverse diameter of the cerebellum percentile in T3 | | 57.50 (60.97, 4.48, 51.80-70.14) | 75.00 (70.82, 1.47, 67.92-73.72) | MWU | 0.029 |
| Child's temperature at birth | | 37.00 (37.09, 0.10, 36.88-37.30) | 37.20 (37.22, 0.04, 37.14-37.29) | T-test | 0.26 |
| Ratio IgM CMV | | 0.31 (0.30, 0.02, 0.26-0.34) | 0.26 (0.28, 0.02, 0.24-0.32) | MWU | 0.25 |
| IgG CMV | | 9950.00 (10913.64, 2034.00, 6683.70-15143.57) | 0.00 (4675.00, 963.19, 2750.23-6599.77) | MWU | < 0.001 |
| Fetal heart rate during labour (FIGO classification) | Pathological % (n) | 28.89 (13) | 10.20 (15) | Chi-sq | < 0.001 |
|  | Suspect % (n) | 8.89 (4) | 28.57 (42) |  |  |
|  | Normal % (n) | 62.22 (28) | 61.22 (90) |  |  |
| Apgar score at 1 minute | | 10.00 (8.73, 0.29, 8.15-9.31) | 10.00 (9.32, 0.11, 9.09-9.54) | MWU | 0.14 |
| Antibiotics | Yes % (n) | 19.35 (12) | 5.62 (10) | Chi-sq | 0.003 |
|  | No % (n) | 80.65 (50) | 94.38 (168) |  |  |
| Real PAPP_A | | 10.04 (19.35, 4.08, 10.90-27.79) | 4.62 (8.76, 1.13, 6.52-11.01) | MWU | 0.004 |
| Ratio of head circumference to femur length in T2 | | 5.04 (5.07, 0.04, 4.99-5.16) | 5.02 (5.06, 0.03, 5.01-5.11) | MWU | 0.99 |
| Duration of the rupture of the membranes (minutes) | | 147.00 (254.22, 41.73, 170.80-337.64) | 222.00 (401.04, 61.59, 279.53-522.54) | MWU | 0.20 |
| Biparietal diameter percentile in T2 | | 49.80 (47.58, 3.08, 41.41-53.75) | 42.85 (43.42, 1.75, 39.97-46.88) | MWU | 0.28 |
| Craniocaudal length percentile in T1 | | 80.00 (82.72, 1.38, 79.91-85.54) | 80.00 (78.92, 1.25, 76.45-81.39) | MWU | 0.27 |
| Origin | African % (n) | 22.22 (14) | 10.05 (19) | Chi-sq | 0.010 |
|  | Caucasians % (n) | 61.90 (39) | 80.42 (152) |  |  |
|  | Other % (n) | 15.87 (10) | 9.52 (18) |  |  |
| Real FBhCG | | 30.55 (45.71, 7.63, 29.93-61.49) | 29.62 (36.76, 2.82, 31.17-42.36) | MWU | 0.46 |
| Ombilical doppler in T3 | | 0.64 (0.63, 0.01, 0.61-0.66) | 0.63 (0.63, 0.01, 0.62-0.64) | T-test | 0.87 |
| PAPP_A (DoE) | | 0.85 (0.83, 0.22, 0.38-1.27) | 0.13 (0.18, 0.08, 0.02-0.33) | MWU | 0.005 |
| Ratio of biparietal diameter to cranio-caudal length in T1 | | 0.34 (0.34, 0.01, 0.33-0.36) | 0.34 (0.35, 0.003, 0.34-0.35) | MWU | 0.54 |
| Biparietal diameter percentile in T3 | | 33.71 (38.47, 4.12, 30.21-46.73) | 35.55 (37.41, 1.90, 33.67-41.15) | MWU | 0.86 |
| FBhCG (DoE) | | 0.47 (0.46, 0.18, 0.10-0.83) | -0.16 (0.06, 0.10, -0.14-0.25) | MWU | 0.011 |
| Fibrinogen in T3 | | 4.85 (4.88, 0.11, 4.66-5.11) | 4.81 (4.84, 0.07, 4.71-4.98) | MWU | 0.93 |
| Platelets T3+DS1 | | 241.00 (251.03, 8.91, 233.20-268.86) × 10^3^ | 245.00 (247.87, 4.74, 238.52-257.23) × 10^3^ | MWU | 0.78 |
| Transverse diameter of the cerebellum percentile in T2 | | 55.00 (50.81, 4.11, 42.41-59.20) | 55.00 (56.59, 1.50, 53.64-59.55) | MWU | 0.32 |
| Number of miscarriages | | 0.00 (0.24, 0.08, 0.08-0.39) | 0.00 (0.46, 0.07, 0.33-0.58) | MWU | 0.044 |
| Ratio of head circumference to femur length in T3 | | 4.74 (4.73, 0.04, 4.65-4.80) | 4.76 (4.77, 0.02, 4.74-4.81) | MWU | 0.26 |
| First mechonium emission (minute) | | 395.00 (439.22, 54.70, 329.35-549.08) | 377.50 (431.80, 34.08, 364.48-499.12) | MWU | 0.74 |
| Abdominal perimeter percentile in T3 | | 50.80 (53.65, 3.53, 46.58-60.72) | 53.19 (52.16, 1.77, 48.66-55.65) | MWU | 0.71 |
| Mother’s body mass index before pregnancy | | 23.40 (25.19, 0.84, 23.51-26.87) | 22.90 (24.41, 0.44, 23.55-25.27) | MWU | 0.44 |
| Weight percentile at birth | | 38.76 (42.39, 3.77, 34.86-49.92) | 39.66 (42.25, 1.99, 38.33-46.17) | MWU | 0.97 |
| Folic Acid | | 0.40 (0.58, 0.16, 0.27-0.90) | 0.40 (0.47, 0.05, 0.36-0.57) | MWU | 0.048 |
| Weight percentile at Day 1 | | 22.86 (33.85, 3.98, 25.86-41.84) | 29.66 (34.81, 1.99, 30.88-38.74) | MWU | 0.57 |
| femur of femur length to cranio-caudal length in T1 | | 0.12 (0.13, 0.01, 0.12-0.14) | 0.13 (0.13, 0.002, 0.12-0.13) | MWU | 0.50 |
| Hemoglobin in T3 | | 11.60 (11.75, 0.14, 11.47-12.03) | 11.80 (11.87, 0.09, 11.69-12.05) | T-test | 0.48 |
| Ratio head circumference to femur length before birth | | 4.64 (4.63, 0.05, 4.54-4.72) | 4.57 (4.61, 0.03, 4.54-4.67) | MWU | 0.56 |
| Ratio of head circumference to height | | 0.70 (0.70, 0.00, 0.69-0.71) | 0.70 (0.70, 0.00, 0.69-0.70) | MWU | 0.35 |
| Abdominal perimeter percentile before birth | | 67.86 (59.19, 4.82, 49.38-69.00) | 53.59 (52.93, 3.08, 46.80-59.05) | MWU | 0.28 |
| Term of birth (days) | | 274.00 (271.51, 2.28, 266.94-276.07) | 274.00 (271.06, 1.07, 268.94-273.18) | MWU | 0.46 |
| Study level | Primary % (n) | 20.63 (13) | 6.35 (12) | Chi-sq | 0.004 |
|  | Secondary % (n) | 53.97 (34) | 60.85 (115) |  |  |
|  | Superior % (n) | 25.40 (16) | 32.80 (62) |  |  |
| Umbilical doppler before birth | | 0.59 (0.58, 0.01, 0.55-0.60) | 0.59 (0.60, 0.01, 0.59-0.62) | T-test | 0.098 |
| Biparietal diameter percentile in T1 | | 50.00 (54.18, 4.44, 45.14-63.22) | 50.00 (54.55, 1.62, 51.36-57.75) | MWU | 0.94 |
| Maternal tobacco use | | 0.00 (1.37, 0.58, 0.21-2.53) | 0.00 (3.06, 0.53, 2.01-4.10) | MWU | 0.019 |
| Head circumference percentile in T3 | | 61.04 (55.36, 3.47, 48.41-62.32) | 55.38 (52.23, 1.70, 48.87-55.59) | MWU | 0.31 |
| Fetal weight estimation percentile in T3 | | 36.68 (43.42, 4.14, 35.13-51.71) | 32.98 (37.11, 1.80, 33.55-40.67) | MWU | 0.26 |
| Newborn feeding | Artificial % (n) | 17.86 (10) | 30.10 (56) | Chi-sq | 0.009 |
|  | Maternal % (n) | 64.29 (36) | 63.98 (119) |  |  |
|  | Mixed % (n) | 17.86 (10) | 5.91 (11) |  |  |
| Head circumference percentile in day 1 | | 47.13 (49.07, 3.58, 41.89-56.24) | 45.62 (47.73, 1.79, 44.20-51.26) | MWU | 0.83 |
| Femur length percentile in T2 | | 53.30 (54.85, 2.97, 48.89-60.81) | 51.60 (51.52, 1.62, 48.32-54.73) | MWU | 0.40 |
| Number of Caesarean sections | | 0.00 (0.19, 0.06, 0.06-0.32) | 0.00 (0.14, 0.03, 0.07-0.20) | MWU | 0.41 |
| Duration of the second part of the labour (minute) | | 14.00 (39.54, 6.88, 25.80-53.28) | 20.00 (55.68, 5.63, 44.58-66.79) | MWU | 0.31 |
| Vitamin D | | 0.00 (29.03, 5.81, 17.41-40.65) × 10^3^ | 0.00 (25.28, 3.27, 18.83-31.73) × 10^3^ | MWU | 0.57 |
| Duration of epidural analgesia (minute) | | 207.00 (205.02, 21.55, 161.93-248.10) | 210.00 (217.29, 14.28, 189.13-245.46) | MWU | 0.85 |
| Apgar score at 3 minutes | | 10.00 (9.44, 0.20, 9.05-9.84) | 10.00 (9.69, 0.07, 9.54-9.83) | MWU | 0.17 |
| First urine (minute) | | 537.50 (555.35, 75.97, 402.82-707.87) | 517.50 (610.65, 43.90, 523.94-697.37) | MWU | 0.45 |
| Pregnancy weight gain | | 13.00 (12.75, 0.74, 11.26-14.25) | 13.00 (12.91, 0.42, 12.08-13.74) | T-test | 0.86 |
| Maternal history of infectious diseases | Yes % (n) | 20.63 (13) | 10.58 (20) | Chi-sq | 0.067 |
|  | No % (n) | 79.37 (50) | 89.41 (169) |  |  |
| Number of urgent Caesarean sections | | 0.00 (0.16, 0.06, 0.05-0.27) | 0.00 (0.10, 0.02, 0.05-0.14) | MWU | 0.43 |
| Skin to skin protocol | Immediate % (n) | 20.63 (13) | 32.45 (61) | Chi-sq | 0.20 |
|  | Precoce % (n) | 19.05 (12) | 15.43 (29) |  |  |
|  | No % (n) | 60.32 (38) | 52.13 (98) |  |  |
| Oxytocin during labor | | 0.00 (7.73, 1.62, 4.49-10.97) | 0.00 (8.22, 0.94, 6.37-10.07) | MWU | 0.59 |
| Fetal cardiac short term variability before birth | | 10.30 (11.84, 0.95, 9.89-13.80) | 10.90 (11.35, 0.35, 10.66-12.03) | MWU | 0.99 |
| Femur length percentile before birth | | 32.81 (38.24, 4.59, 28.90-47.58) | 28.42 (33.57, 2.97, 27.67-39.47) | MWU | 0.35 |
| Familal maternal history of endocrine diseases | Yes % (n) | 41.27 (26) | 38.62 (73) | Chi-sq | 0.82 |
|  | No % (n) | 58.73 (37) | 61.38 (116) |  |  |
| Fetal weight estimation percentile before birth | | 89.79 (71.35, 5.80, 59.54-83.15) | 77.34 (62.11, 4.05, 54.07-70.16) | MWU | 0.17 |
| Instrumental delivery | VB % (n) | 66.67 (42) | 63.49 (120) | Chi-sq | 0.89 |
|  | Cesar % (n) | 23.81 (15) | 24.87 (47) |  |  |
|  | Forceps % (n) | 4.76 (3) | 7.41 (14) |  |  |
|  | Ventouse % (n) | 4.76 (3) | 4.23 (8) |  |  |
| Serology of toxoplasmaosisa | Immunized % (n) | 31.75 (20) | 34.92 (66) | Chi-sq | 0.76 |
|  | Negative % (n) | 68.25 (43) | 65.08 (123) |  |  |
| Placenta Grannum classification in T3 | | 1.00 (0.97, 0.13, 0.70-1.24) | 1.00 (0.94, 0.05, 0.84-1.04) | MWU | 0.79 |
| Biparietal diameter percentile before birth | | 38.20 (48.03, 5.28, 37.29-58.77) | 33.71 (38.77, 3.11, 32.59-44.95) | MWU | 0.13 |
| Femur length percentile in T1 | | 50.00 (49.74, 4.08, 41.41-58.07) | 50.00 (51.52, 1.44, 48.68-54.36) | MWU | 0.61 |
| Glycemia at birth | | 0.65 (0.66, 0.03, 0.59-0.72) | 0.63 (0.66, 0.02, 0.62-0.69) | T-test | 0.99 |
| Number of scheduled Caesarean sections | | 0.00 (0.03, 0.02, -0.01-0.08) | 0.00 (0.04, 0.02, 0.01-0.08) | MWU | 0.99 |
| Fetal cardiac rhythm FIGO 2 groups | Normal % (n) | 62.22 (28) | 61.22 (90) | Chi-sq | 0.96 |
|  | Pathological % (n) | 37.78 (17) | 38.78 (57) |  |  |
| Foot length in T3 | | 67.00 (69.40, 1.51, 65.97-72.83) | 65.50 (65.39, 1.11, 63.11-67.68) | T-test | 0.046 |
| Pathological pH | Normal % (n) | 82.46 (47) | 90.76 (167) | Chi-sq | 0.13 |
|  | Pathological % (n) | 17.54 (10) | 9.24 (17) |  |  |
| Maternal history of endocrine diseases | Yes % (n) | 14.29 (9) | 12.70 (24) | Chi-sq | 0.91 |
|  | No % (n) | 85.71 (54) | 87.30 (165) |  |  |
| Familial paternal history of endocrine diseases | Yes % (n) | 10.34 (6) | 15.30 (28) | Chi-sq | 0.47 |
|  | No % (n) | 89.66 (52) | 84.70 (155) |  |  |
| Controlled diabetes | Yes % (n) | 10.71 (6) | 14.86 (26) | Chi-sq | 0.73 |
|  | No % (n) | 8.93 (5) | 8.00 (14) |  |  |
|  | NS % (n) | 80.36 (45) | 77.14 (135) |  |  |
| Size of lateral ventricle percentile in T3 | | 3.00 (12.10, 4.54, 2.71-21.50) | 3.00 (6.81, 1.19, 4.45-9.18) | MWU | 0.33 |
| Antibiotics during labour | Yes % (n) | 15.87 (10) | 22.75 (146) | Chi-sq | 0.33 |
|  | No % (n) | 84.13 (53) | 77.25 (43) |  |  |
| Apgar score at 5 minutes | | 10.00 (9.67, 0.16, 9.35-9.98) | 10.00 (9.86, 0.06, 9.74-9.97) | MWU | 0.37 |
| Hearing test symmetry | Symmetric % (n) | 91.49 (43) | 93.39 (113) | Chi-sq | 0.92 |
|  | Asymmetric % (n) | 8.51 (4) | 6.61 (8) |  |  |
| Hearing test | Normal % (n) | 78.26 (36) | 80.17 (97) | Chi-sq | 0.95 |
|  | Abnormal % (n) | 21.74 (10) | 19.83 (24) |  |  |
| Treatment for labor induction | Yes % (n) | 20.63 (13) | 22.75 (43) | Chi-sq | 0.86 |
|  | No % (n) | 70.37 (50) | 77.25 (146) |  |  |
| Type of delivery | Vaginal % (n) | 69.84 (44) | 73.02 (138) | Chi-sq | 0.75 |
|  | Caesarean % (n) | 30.16 (19) | 26.98 (51) |  |  |
| Coagulation in T3 | Normal % (n) | 89.83 (53) | 88.34 (144) | Chi-sq | 0.95 |
|  | Abnormal % (n) | 10.17 (6) | 11.66 (19) |  |  |
| Streptococcus B vaginal swab | Positive % (n) | 10.53 (6) | 7.22 (13) | Chi-sq | 0.60 |
|  | Negative %(n) | 89.47 (51) | 92.78 (167) |  |  |
| Apgar score at 10 minutes | | 10.00 (9.78, 0.11, 9.55-10.00) | 10.00 (9.91, 0.04, 9.83-9.99) | MWU | 0.074 |
| Maternal history of auto-immune diseases | Yes % (n) | 23.81 (15) | 19.05 (36) | Chi-sq | 0.53 |
|  | No % (n) | 76.19 (48) | 80.95 (153) |  |  |
| Gestational diabetes | Yes % (n) | 16.07 (9) | 19.43 (34) | Chi-sq | 0.72 |
|  | No % (n) | 83.93 (47) | 80.57 (141) |  |  |
| Aspegic | Yes % (n) | 1.61 (1) | 8.99 (16) | Chi-sq | 0.096 |
|  | No % (n) | 98.39 (61) | 91.01 (162) |  |  |
| Rh blood group system | Positive % (n) | 84.13 (53) | 85.19 (161) | Chi-sq | 1.00 |
|  | Negative % (n) | 15.87 (10) | 14.81 (28) |  |  |
| IgM | Positive % (n) | 4.55 (1) | 0 (0) | Chi-sq | 0.59 |
|  | Negative % (n) | 95.45 (21) | 100 (61) |  |  |
| Corticosteroids | Yes % (n) | 6.45 (4) | 7.30 (13) | Chi-sq | 0.95 |
|  | No % (n) | 93.55 (58) | 92.70 (165) |  |  |
| Familial paternal history of auto-immune diseases | Yes % (n) | 6.90 (4) | 4.37 (8) | Chi-sq | 0.67 |
|  | No % (n) | 93.10 (54) | 95.63 (175) |  |  |
| Paternal history of endocrine diseases | Yes % (n) | 0 (0) | 0.55 (1) | Chi-sq | 0.54 |
|  | No % (n) | 100 (58) | 99.45 (182) |  |  |
| Paternal history of infectious diseases | Yes % (n) | 1.72 (1) | 0 (0) | Chi-sq | 0.54 |
|  | No % (n) | 98.28 (57) | 100 (183) |  |  |
| Paternal history of auto-immune diseases | Yes % (n) | 3.45 (2) | 2.19 (4) | Chi-sq | 0.96 |
|  | No % (n) | 96.55 (56) | 97.81 (179) |  |  |
| Medically assisted procreation | Yes % (n) | 6.35 (4) | 12.70 (24) | Chi-sq | 0.25 |
|  | No % (n) | 93.65 (59) | 87.30 (165) |  |  |
| Serology of rubella | Immunized % (n) | 92.06 (58) | 94.18 (178) | Chi-sq | 0.76 |
|  | Negative % (n) | 7.94 (5) | 5.82 (11) |  |  |
