# Supplementary material for: Machine learning analysis of pregnancy data enables early identification of a subpopulation of newborns with ASD
Source: Sci Rep. 2021 Mar 25;11:6877. doi: 10.1038/s41598-021-86320-0 (PMC7994821; doi:10.1038/s41598-021-86320-0)
Supplement: Supplementary file 6 — Supplementary Table S4. [file 41598_2021_86320_MOESM6_ESM.docx]

# **Supplementary Table S4**

**Machine learning analysis of pregnancy data enables early identification of a subpopulation of newborns with ASD**

Hugues Caly ^1+^, Hamed Rabiei ^2,3+^, Perrine Coste-Mazeau ^1^, Sebastien Hantz ^4,5^, Sophie Alain ^4,5^, Jean-Luc Eyraud ^1^, Thierry Chianea ^6^, Catherine Caly ^1^, David Makowski ^7^, Nouchine Hadjikhani ^8,9^, Eric Lemonnier ^10^, Yehezkel Ben-Ari ^2,3*^

1. Gynecology-Obstetrics Department, Mère-Enfant Hospital, University Hospital Center, Limoges, France

2. BABiomedical, Luminy Scientific Campus, Marseille, France

3. Neurochlore, Luminy Scientific Campus, Marseille, France

4. Bacteriology-Virology-Hygiene Department, University Hospital Center, Limoges, France

5. French National Reference Center for Herpes Viruses, University Hospital Center, Limoges, France

6. Department of Biochemistry and Molecular Genetics, Dupuytren University Hospital, Limoges, France

7. INRAE, UMR MIA 518 INRA AgroParisTech Université Paris-Saclay, Paris, France

8. Martinos Center for Biomedical Imaging, Harvard Medical School, Boston, USA

9. Gillberg Neuropsychiatry Center, Sahlgrenska Academy, Gothenburg University, Sweden

10. Autism Expert Center and Autism Resource Center of Limousin, University Hospital Center, Limoges, France

+ Equally contributing authors

* Corresponding author

**Address for correspondence**

Yehezkel Ben-Ari

Neurochlore

Bâtiment Beret Delaage, Case 922

Zone Luminy Entreprises Biotech, 163 Avenue de Luminy, 13288 Marseille Cedex 09, France

[ben-ari@neurochlore.fr](mailto:ben-ari@neurochlore.fr)

**Table S4.** Complete list of all features collected from the 1^st^, 2^nd^ and 3^rd^ trimester, before birth, and during the post-natal period.

| **List of collected features** | | | |
| --- | --- | --- | --- |
| **General information about parents** | | | |
| - Mother’s age - Father’s age - Family history of mother and father (endocrine and autoimmune) - History of mother and father (endocrine, infectious, and autoimmune) | - Maternal body mass index before pregnancy - Maternal serologies for rubella, toxoplasmosis and CMV, - Maternal blood RH type | | - Maternal tobacco use - Ethnic origin - Level of study - Number of pregnancies - Number of miscarriages - Number of emergency or scheduled Caesarean sections |
| **Treatments during pregnancy** | | | |
| - Vitamin D - Folic acid - Antibiotics | | - Aspirin - Corticosteroids | |
| **General pregnancy data** | | | |
| - Real first trimester marker (Actual HT21 rate) - Gestational diabetes - Controlled gestational diabetes - Weight gain during pregnancy | | - PAPP-A real dosage and DoE - FBhCG real dosage and DoE - Timing of fetal rotation on head | |
| **First trimester of pregnancy** | | | |
| - Nuchal translucency measurement and percentile - Biparietal diameter measurement and percentile - Femur length measures and percentile | - Cranio-caudal length measurement and percentile - Biparietal diameter to cranio-caudal length ratio - Femur length measurement and percentile | | - Femoral length to cranio-caudal ratio |
| **Second trimester of pregnancy** | | | |
| - Biparietal diameter measurement and percentile - Femur length measurement and percentile - Abdominal perimeter measurement and percentile | - Head circumference measurement and percentile - Ratio of head circumference to femoral length - Estimation of fetal weight measurement and percentile | | - Transverse diameter of the cerebellum measurement and percentile - Lateral ventricle measurement and percentile - Nasal bone length - Foot length |
| **Third trimester of pregnancy** | | | |
| - Biparietal diameter measurement and percentile - Femur length measurement and percentile - Abdominal perimeter measurement and percentile - Umbilical Doppler | - Head circumference measurement and percentile - Ratio of head circumference to femoral length - Estimation of fetal weight measurement and percentile | | - Transverse diameter of the cerebellum measurement and percentile - Lateral ventricle measurement and percentile - Nasal bone length - Foot length - Placenta Grannum classification |
| **Prepartum phase** | | | |
| - Biparietal diameter measurement and percentile - Femur length measurement and percentile - Abdominal perimeter measurement and percentile - Head circumference measurement and percentile | - Ratio of head circumference to femoral length - Umbilical Doppler - Short-term variability in fetal heart rate - Estimation of fetal weight measurement and percentile | | - Streptococcus B vaginal swab - White cells - Hemoglobin - Platelets - Coagulation with fibrinogenemia |
| **Birth** | | | |
| - Term of birth - Type of delivery - Treatment for labor induction - Antibiotic during labor - FIGO stage of fetal heart rate during labor | - Duration of the first and second part of the labor - Duration of the rupture of the membranes | | - Duration of epidural analgesia - Use of oxytocin in labor maintenance |
| **Child** | | | |
| - Sex - Apgar score at 1, 3, 5 and 10 minutes - Birth weight and percentile - Temperature of the child at birth - Skin-to-skin protocol - Glycemia at birth | - pH and pCO2 on arterial blood at the umbilical cord - Weight and percentile at D1 - Delta weight and percentile between birth and D1 - Head circumference measures and percentile at day 1 | | - Head circumference to size ratio - Hearing test - Type of breastfeeding - Mechonium emission - Emission of first urine - Temperature at D1 - Delta temperature between birth and D1 |
